# RNA Binding by the *Campylobacter jejuni* Post-transcriptional Regulator CsrA

**DOI:** 10.3389/fmicb.2019.01776

**Published:** 2019-08-07

**Authors:** Faiha M. El Abbar, Jiaqi Li, Harry C. Owen, C. Luke Daugherty, Claudia A. Fulmer, Marek Bogacz, Stuart A. Thompson

**Affiliations:** Division of Infectious Diseases, Department of Medicine, Augusta University, Augusta, GA, United States

**Keywords:** motility, flagella, biofilm, regulation, flagellin

## Abstract

*Campylobacter jejuni* is a Gram-negative rod-shaped bacterium that commensally inhabits the intestinal tracts of livestock and birds, and which also persists in surface waters. *C. jejuni* is a leading cause of foodborne gastroenteritis, and these infections are sometimes associated with the development of post-infection sequelae such as Guillain-Barré Syndrome. Flagella are considered a primary virulence factor in *C. jejuni,* as these organelles are required for pathogenicity-related phenotypes including motility, biofilm formation, host cell interactions, and host colonization. The post-transcriptional regulator CsrA regulates the expression of the major flagellin FlaA by binding to *flaA* mRNA and repressing its translation. Additionally, CsrA has previously been shown to regulate 120–150 proteins involved in diverse cellular processes. The amino acid sequence of *C. jejuni* CsrA is significantly different from that of *Escherichia coli* CsrA, and no previous research has defined the amino acids of *C. jejuni* CsrA that are critical for RNA binding. In this study, we used *in vitro* SELEX to identify the consensus RNA sequence mAwGGAs to which *C. jejuni* CsrA binds with high affinity. We performed saturating site-directed mutagenesis on *C. jejuni* CsrA and assessed the regulatory activity of these mutant proteins, using a reporter system encoding the 5′ untranslated region (5′ UTR) upstream of *flaA* linked translationally to the *C. jejuni astA* gene. These assays allowed us to identify 19 amino acids that were involved in RNA binding by CsrA, with many but not all of these amino acids clustered in predicted beta strands that are involved in RNA binding by *E. coli* CsrA. Decreased *flaA* mRNA binding by mutant CsrA proteins L2A and A36V was confirmed by electrophoretic mobility shift assays. The majority of the amino acids implicated in RNA binding were conserved among diverse *Campylobacter* species.

## Introduction

*Campylobacter jejuni* is a leading bacterial cause of foodborne gastroenteritis throughout the world (WHO, 2015), with 1.3 million cases of *Campylobacter* infections in the US (Tack et al., 2019) and 96 million cases globally each year (WHO, 2013). Symptoms typically consist of 4–7 days of severe watery to bloody diarrhea, abdominal cramping, fever, vomiting, and dehydration (Kaakoush et al., 2015). *C. jejuni* infection is generally acute and self-limiting, but in some patients it is associated with the development of post-infection sequelae such as autoimmune-mediated Guillain-Barré Syndrome, the leading cause of acute paralysis (Nachamkin et al., 2000). *C. jejuni* commensally colonizes the gastrointestinal tract of animals including poultry, cattle, swine, and sheep (Kaakoush et al., 2015). Therefore, the source of infection is often the consumption of contaminated meat (especially poultry) or drinking of contaminated raw milk (Kaakoush et al., 2015). However, exposure to environmental sources such as surface waters is suggested to cause a large proportion of *Campylobacter* infections (Champion et al., 2005). To survive in diverse hosts and environmental niches, *C. jejuni* must accommodate a range of stresses such as changes in temperature, pH, oxygen level, and exposure to host bile, digestive enzymes, and inflammatory responses. Flagella are well-characterized virulence factors in *C. jejuni* as they are required for pathogenicity-related phenotypes including colonization (Wassenaar et al., 1993), interactions with host cells (Guerry, 2007; Freitag et al., 2017), biofilm formation (Svensson et al., 2014), and the secretion of virulence-associated proteins such as Cia invasion antigens (Konkel et al., 1999). Mutants lacking flagella are highly attenuated in animal models (Guerry, 2007). Flagellar filaments are composed primarily of the major flagellin FlaA, the expression of which is regulated transcriptionally by FlgSR, σ^54^, and σ^28^ (Lertsethtakarn et al., 2011), as well as post-transcriptionally by the RNA-binding protein CsrA (carbon storage regulator A) (Fields and Thompson, 2008; Dugar et al., 2016; Fields et al., 2016). A *C. jejuni csrA* mutant shows significant reduction in epithelial cell adherence, resistance to oxidative stress, motility, biofilm formation, and ability to colonize mice, as well as a paradoxically increased ability to invade host cells (Fields and Thompson, 2008; Fields et al., 2016). Consistent with these phenotypes, a *C. jejuni csrA* mutant exhibited dysregulation of 120–150 proteins involved in motility, chemotaxis, host cell adherence and invasion, oxidative stress resistance, TCA cycle, respiration, and amino acid and acetate metabolism (Fields et al., 2016; Li et al., 2018). This suggests the importance of CsrA as a major global regulatory protein in *C. jejuni.*

In *Escherichia coli* and other studied bacteria, CsrA is a homodimeric protein, with each subunit composed of five beta (β) strands (β_1_–β_5_). Two identical RNA-binding pockets are formed by β_1_ and β_5_ of opposite subunits (Mercante et al., 2006, 2009; Romeo et al., 2013; Altegoer et al., 2016). CsrA typically binds the 5′ untranslated region (5′ UTR) at one or more sites of its target mRNAs, often at or near the ribosome-binding site (RBS), and usually at a stem-loop containing a conserved AnGGA sequence motif within the hairpin (Romeo and Babitzke, 2018). Binding of CsrA to mRNA blocks ribosome access and represses the initiation of translation, but it can also influence mRNA stability (Romeo and Babitzke, 2018). Regulation of CsrA activity is mediated in *E. coli* and other bacteria by competitive binding to small RNAs (e.g., *csrB*, *csrC*). These sRNAs contain many CsrA-binding sites which sequester CsrA and titrate its binding to target mRNAs (Romeo and Babitzke, 2018). However, *C. jejuni* lacks these antagonizing sRNAs, and CsrA activity is instead regulated by a mechanism similar to that of *Bacillus subtilis* where upon secretion of the major flagellin (FlaA), the flagellar chaperone FliW is released and binds its alternate partner CsrA (Mukherjee et al., 2011, 2016; Dugar et al., 2016; Radomska et al., 2016; Li et al., 2018). Binding to FliW modulates CsrA binding to target mRNAs and alleviates CsrA repression of flagellin expression, a regulatory mechanism required for proper flagellar morphogenesis (Mukherjee et al., 2011; Dugar et al., 2016; Li et al., 2018).

In *C. jejuni,* CsrA binds *flaA* mRNA and directly represses its translation (Dugar et al., 2016; Fields et al., 2016; Radomska et al., 2016). Although a *C. jejuni csrA* mutant shows normal flagellar structure (Fields et al., 2016), the decreased motility of the *csrA* mutant (Fields and Thompson, 2008) suggests that regulation of FlaA expression by CsrA is required for proper motility. The *E. coli* and *C. jejuni* CsrA proteins have significant divergence in amino acid sequence (Fields and Thompson, 2012), raising the question of whether features of RNA binding that were determined for *E. coli* also apply to *C. jejuni*. *C. jejuni* CsrA complements an *E. coli csrA* mutant for some, but not all, phenotypes (Fields and Thompson, 2012), suggesting some divergence of its RNA-binding characteristics. In contrast to *E. coli* CsrA, there have been no previous studies defining the amino acids of *C. jejuni* CsrA that are critical for RNA binding. Understanding the mechanism by which CsrA interacts with *flaA* mRNA may help in future development of strategies to overcome the impact of *C. jejuni* infection. In addition, the mechanism of *flaA* mRNA-CsrA interaction could serve as a model for *C. jejuni* CsrA interaction with other important target mRNAs (Fields et al., 2016; Li et al., 2018). In this study, we identified the consensus RNA sequence to which CsrA binds with high affinity, and determined the amino acid residues of CsrA that are critical for *flaA* mRNA binding.

## Materials and Methods

### Bacterial Strains, Growth Conditions, Plasmids, and PCR Primers

The bacterial strains and plasmids used in this study are listed in Table 1. All *E. coli* strains were grown at 37°C in Luria-Bertani (LB) broth or plates. When appropriate, growth media were supplemented with ampicillin (amp; 100 μg/ml) or chloramphenicol (cm; 30 μg/ml). *C. jejuni* strain 81–176 was used as a source of chromosomal DNA and was grown on Mueller-Hinton (MH) agar at 42°C in a tri-gas incubator (85% N_2_, 10% CO_2_, 5% O_2_). PCR primers are listed in Supplementary Table S1.

**Table 1:** Bacterial strains and plasmids used in this study.

Strain or plasmid	Description	Resistance	Source or reference
**Strain**			
*Campylobacter jejuni*			
81–176	Wild type		(Black et al., 1988)
*Escherichia coli*			
JM109	Cloning strain		Promega
One shot top 10	Cloning strain		Thermo
DH5α	Cloning strain		Thermo
BL21(DE3)	Protein expression strain		Promega
BL21(DE3)pLysS	Protein expression strain	cm	Promega
**Plasmids**			
pCRII-TOPO	Cloning vector	amp, km	Invitrogen
pCR2.1-TOPO	Cloning vectr	amp, km	Invitrogen
pET-20b(+)	Cloning vector	amp	Novagen
pET-20b-CsrA	*csrA* cloned into pET-20b(+)	amp	(Fields et al., 2016)
pACYC184	Cloning vector	cm	NEB
pFE101	*flaA* 5′ UTR cloned into pCR2.1-TOPO	amp, km	This work
pFE102	*astA* cloned into pFE101	amp, km	This work
pJOFE	*flaA*-*astA* translational reporter in pACYC184	cm	This work

### *In vitro* Systematic Evolution of Ligands by Exponential Enrichment

We performed *in vitro* systematic evolution of ligands by exponential enrichment (SELEX) (Tuerk and Gold, 1990) as modified by (Dubey et al., 2005), using purified *C. jejuni* CsrA-His_6_ (see below). Briefly, we first created a DNA template by synthesizing an 81-base oligonucleotide (SELEX15) consisting of a randomized 15-mer (N_15_, where *N* = any nucleotide) flanked by two constant regions (Supplementary Table S1). PCR on the SELEX15 template using primers P1 and P2 (Supplementary Table S1) yielded a complex mixture of 81-bp DNA fragments (a total of ~1 × 10^5^ molecules containing every possible sequence of the random central region), which was used for *in vitro* transcription. Template DNA was removed by DNase I treatment, and transcribed RNA was mixed with *C. jejuni* CsrA-His_6_. CsrA-His_6_-RNA complexes were affinity purified using Ni-NTA slurry. Bound RNA was purified *via* phenol:chloroform extraction and converted to cDNA. The selected templates were then subjected to a total of 10 rounds of PCR amplification and selection as described above. The progress of the selection process was monitored by using gel mobility shift analysis, observing an increasing ability of *C. jejuni* CsrA-His_6_ to retard the mobility of the affinity-selected RNA pools. A total of 57 RT-PCR products from rounds nine and ten were cloned and sequenced; 51 unique sequences were used to generate a consensus *C. jejuni* CsrA-binding sequence following alignment using Clustal Omega at EMBL-EBI (Sievers et al., 2011; Li et al., 2015). The predicted secondary structure for each sequence was also assessed using MFOLD (Zuker, 2003).

### Site-Directed Mutagenesis of *csrA*

Site-directed mutagenesis (SDM) was performed with a Q5 SDM kit (NEB, Ipswich, MA) using the primers listed in Supplementary Table S1. Plasmid pET-20b-CsrA (Fields et al., 2016) was used as PCR template. Each CsrA amino acid was changed individually to alanine, except for two native alanine residues (A30 and A36) that were changed to valine. The first methionine was also substituted with alanine, but an additional methionine was added upstream of the M1A mutation to initiate protein translation. The pET-20b plasmids containing *csrA* point mutations were all verified by DNA sequencing.

### Construction of a Translational Reporter System

For assessing *flaA* mRNA binding by CsrA, we designed a translational reporter by cloning DNA encoding the *flaA* 5′ UTR upstream of the assayable *C. jejuni* gene *astA* encoding arylsulfatase (Yao and Guerry, 1996; Hendrixson and DiRita, 2003). DNA encoding the *flaA* 5′ UTR was synthesized and cloned downstream of the *lac* promoter in pCR2.1-TOPO by a commercial vendor (IDT, Coralville, IA), yielding plasmid pFE101 (Table 1). Inverse PCR was performed on pFE101 to introduce an NdeI site downstream of the *flaA* 5′ UTR DNA using primers FME01 and FME02 (Supplementary Table S1). The *astA* reporter gene was amplified from *C. jejuni* 81–176 chromosomal DNA using the primers FME03 and FME04 (Supplementary Table S1), and cloned downstream of the *flaA* 5′ UTR DNA using the restriction enzymes NdeI and NotI, resulting in plasmid pFE102 (Table 1). Inverse PCR using primers JO-4 and JO-5 (Supplementary Table S1) was performed on pFE102 (containing the *flaA* 5′ UTR translationally linked to *astA*, under control of the *lac* promoter) to introduce a SalI site upstream of the *lac* promoter for subcloning purposes. The SalI fragment of pFE102 was then ligated with SalI-digested pACYC184 to yield pJOFE (Table 1). *E. coli* BL21(DE3) cells were transformed with pJOFE and pET-20b expressing WT CsrA, CsrA with the aforementioned point mutations, or pET-20b alone (negative control). Expression of AstA from the translational reporter was assessed in two ways. Plates used to recover transformed cells contained 50 μg/ml of arylsulfatase substrate (5-bromo-4-chloro-3-indolyl sulfate potassium salt; Millipore-Sigma, St. Louis, MO). The intensity of blue color of colonies on these plates reflected the degree of AstA expression. To quantify AstA activity, we used an arylsulfatase assay (Hendrixson and DiRita, 2003). Briefly, this assay quantifies the AstA-mediated conversion of the substrate nitrophenylsulfate to nitrophenol, which is measured by absorbance at 410 nm. Results were analyzed using one-way ANOVA in GraphPad Prism (GraphPad Software, Inc.), with Dunnett’s multiple comparisons test, using *p* < 0.05 to indicate significance. To verify expression of CsrA in *E. coli*, the samples used in the arylsulfatase assay were tested in western blots using CsrA-specific polyclonal antiserum (antibody dilution 1:1,000) (Fields et al., 2016). Experiments were done a minimum of three times, using triplicate samples.

### Purification of Wild Type and Mutant CsrA-His_6_ Proteins

Wild type and mutants of CsrA (L2A) and (A36V) with C-terminal His_6_-tag were overexpressed in *E. coli* BL21(DE3) pLysS cells. Cells were grown in LB broth at 37°C until they reached an OD_600_ of 0.6, and protein expression was subsequently induced with 0.5 mM IPTG and carried out at 20°C overnight. Cells were disrupted in extraction buffer (50 mM Tris–HCl pH 8.5, 1 M NaCl, 20 mM imidazole, 10% glycerol) with a French press (Thermo Fisher Scientific). The lysate was cleared by centrifugation (15,000 × *g*) and mixed with Ni-NTA chromatography resin (Ni-NTA Agarose, Qiagen). After protein binding (1 h in 4°C), the resin was washed three times with 10 resin volumes of extraction buffer. The protein was eluted with 50 mM Tris–HCl, pH 8.5, 1 M NaCl, 250 mM imidazole, 10% glycerol, and dialyzed into 20 mM sodium phosphate pH 7.5, 150 mM NaCl. The final CsrA protein sample was obtained by gel filtration on Superdex 75 10/300 column (GE Healthcare) in the same buffer.

### Electrophoretic Mobility Shift Assay

Electrophoretic mobility shift assay (EMSA) experiments were performed as described previously (Yakhnin et al., 2012; Fields et al., 2016), using purified *C. jejuni* CsrA(WT)-His_6_, CsrA(L2A)-His_6_, and CsrA(V36A)-His_6_. PCR using 81–176 chromosomal DNA and primers containing a T7 promoter sequence (Fields et al., 2016) was performed to generate *flaA* 5′ UTR DNA templates to be used for *in vitro* transcription. An *E. coli phoB* 5′ UTR DNA template was generated to be used as a CsrA-non-binding control, as described (Patterson-Fortin et al., 2013; Fields et al., 2016). RNA was synthesized using a MEGAscript™ T7 Transcription kit (Ambion), and purified *via* phenol:chloroform extraction. Purified RNAs were end-labeled with ^32^P using a KinaseMax™ 5′ End-Labeling kit (Ambion). Radiolabeled RNA at a concentration of 1 nM was then incubated with different concentrations (0–4 μM) of purified CsrA-His_6_ (WT, L2A or A36V) in binding reactions. Samples were resolved on 12% native polyacrylamide gels and visualized on a phosphorimager.

## Results

### *In vitro* Systematic Evolution of Ligands by Exponential Enrichment Defines High-Affinity RNA Ligands Recognized by *C. jejuni* CsrA

**Figure 1:**
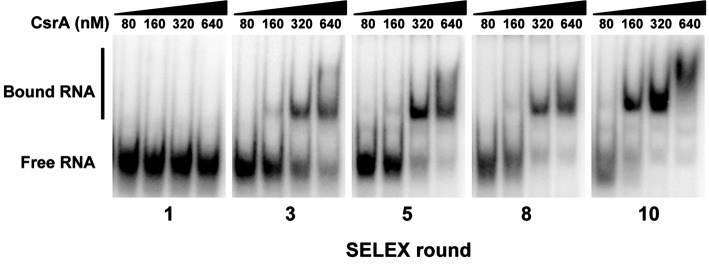
Identification of high-affinity RNA ligands for *C. jejuni* CsrA using *in vitro* SELEX. A complex mixture of oligonucleotides was designed containing PCR primers flanking a random N_15_ central region. RNAs transcribed *in vitro* from this mixture were bound to CsrA-His_6_, purified, converted to cDNA, then the enriched pool was subsequently used in a total of 10 rounds of SELEX. To monitor the progress of SELEX, gel shift assays were performed using increasing concentrations of purified CsrA-His_6_ and enriched RNAs from cycles 1, 3, 5, 8, and 10. Increased conversion of unbound RNA (“Free RNA”) to enriched CsrA-bound RNA ligands (“Bound RNA”) was visible in successive rounds of SELEX.

**Figure 2:**
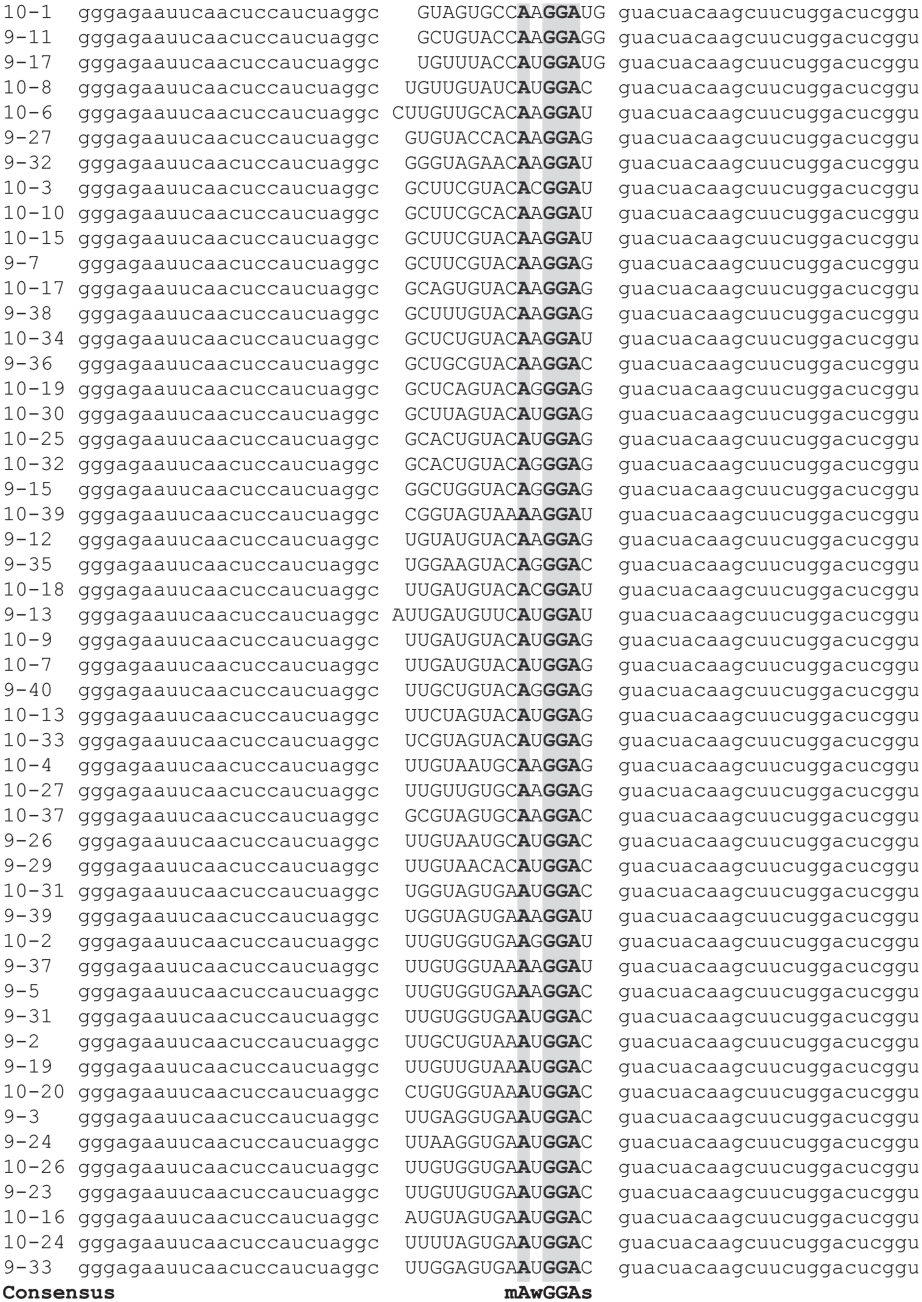
Alignment of SELEX-derived RNA templates. Following nine and ten cycles of enrichment, cDNAs corresponding to high-affinity *C. jejuni* CsrA RNA ligands were sequenced and aligned using Clustal Omega (Li et al., 2015) to generate the consensus binding sequence mAwGGAs. Gray shading indicates nucleotides present in every SELEX ligand.

**Figure 3:**
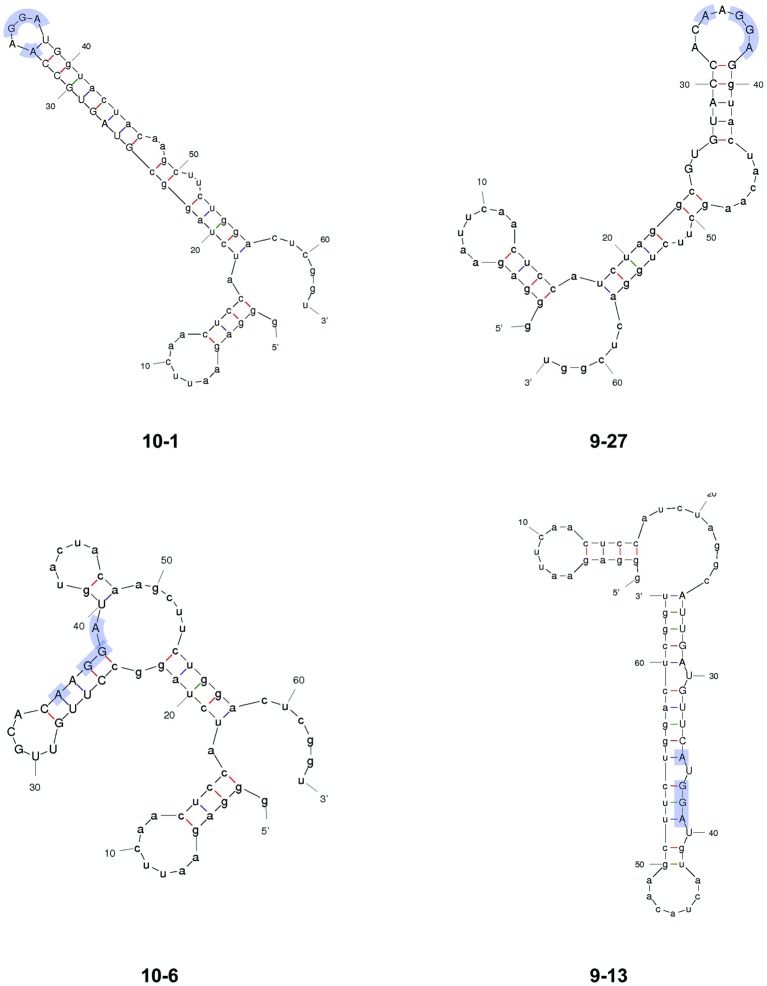
Predicted secondary structures of representative SELEX-enriched CsrA-binding RNAs. Selected RNAs from SELEX were folded using MFOLD. RNAs 10–1 and 9–27 represent the majority of enriched RNAs, in which the AnGGA motif (blue shading) was present at the end of long stem-loops. In RNAs 10–6 and 9–13, the AnGGA motifs were present within the stems instead.

The consensus binding sequence of *E. coli* CsrA was determined previously and shown to be RUACARGGAUGU (Dubey et al., 2005). However, the RNA-binding regions of *C. jejuni* CsrA homologous to those of *E. coli* CsrA (Mercante et al., 2006) differ somewhat in primary amino acid sequence (Fields and Thompson, 2012), suggesting the possibility that the RNA sequence to which *C. jejuni* CsrA binds is also somewhat divergent. Consequently, we employed *in vitro* SELEX to identify high-affinity RNA ligands to which *C. jejuni* CsrA binds. A total of 10 rounds of amplification and affinity purification were used to generate enriched RNA molecules that bound CsrA with increasing affinity, which was measured by gel shift assays (Figure 1). At nine and ten rounds, bound RNAs were converted to cDNA, cloned, and sequenced. Alignment of the sequences (Figure 2) revealed the following features. The deduced binding site was mAwGGAs, in which the nucleotides A and GGA were present in every selected ligand. The first nucleotide in this consensus sequence was either C (67%) or A (33%) (ambiguity code “m”). The nucleotide immediately preceding the conserved GGA motif was A or U (ambiguity code “w”) in 43/51 ligands (84%). Following the GGA trinucleotide, G or C (ambiguity code “s”) occurred in 37/51 ligands (73%). In each of the CsrA-binding sequences that were enriched in these experiments, the sites were present in the 3′ half of the randomized nucleotide region. Using MFOLD secondary structure predictions, in 49 of the 51 unique sequences the A_GGA motif was present within hairpins of long stem-loops (Figure 3, Supplementary Figure S1). Because the A_GGA motif was generally at positions 9–13 of the randomized nucleotide region, nucleotides 1–8 typically were complementary to PCR primer P2 so as to form stable stems flanking the GGA-containing loops. However, in sequences 10–6 and 9–13, the A_GGA motifs were predicted to be present in stems rather than in loops (Figure 3).

### Multiple Amino Acids Are Involved in the Interaction of CsrA With *flaA* 5′ UTR

**Figure 4:**
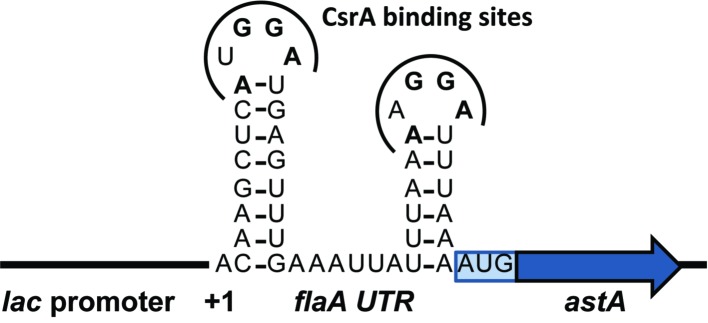
Schematic of translational reporter construct pJOFE. The *C. jejuni* reporter gene *astA* was cloned downstream of DNA encoding the *flaA* 5′ UTR to create a translational fusion under the control of the *E. coli lac* promoter, then the construct was cloned into pACYC184 to yield pJOFE. The 5′ UTR of *flaA* is predicted to fold into two stem-loops with two CsrA-binding sites containing the A(U/A)GGA motif.

**Figure 5:**
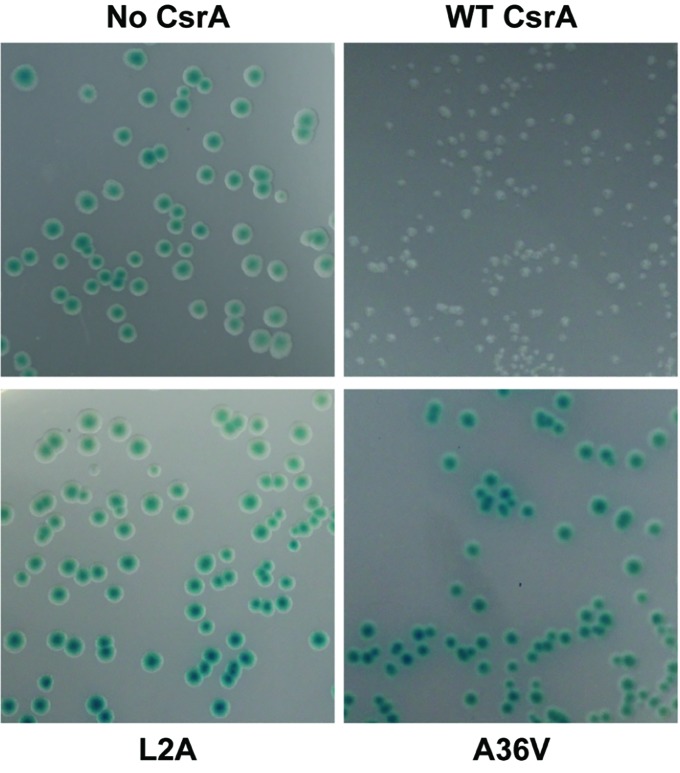
Repression of AstA translational fusion by WT and mutant CsrA proteins. *E. coli* BL21(DE3) was co-transformed with the translational reporter pJOFE (encoding the *flaA* 5′ UTR translationally linked to *astA*, under control of the *lac* promoter) and either: pET-20b (“No CsrA”, top left panel), pET-20b-CsrA (“WT CsrA”, top right panel), pET-20b- CsrA-L2A (“L2A”, bottom left panel), or pET-20b-CsrA-A36V (“A36V”, bottom right panel), and plated on LB plates containing 50 μg/ml of arylsulfatase substrate. The intensity of the blue color of the colonies indicates AstA enzyme activity and lack of CsrA regulatory activity. Experiments were done a minimum of three times, using triplicate samples.

**Figure 6:**
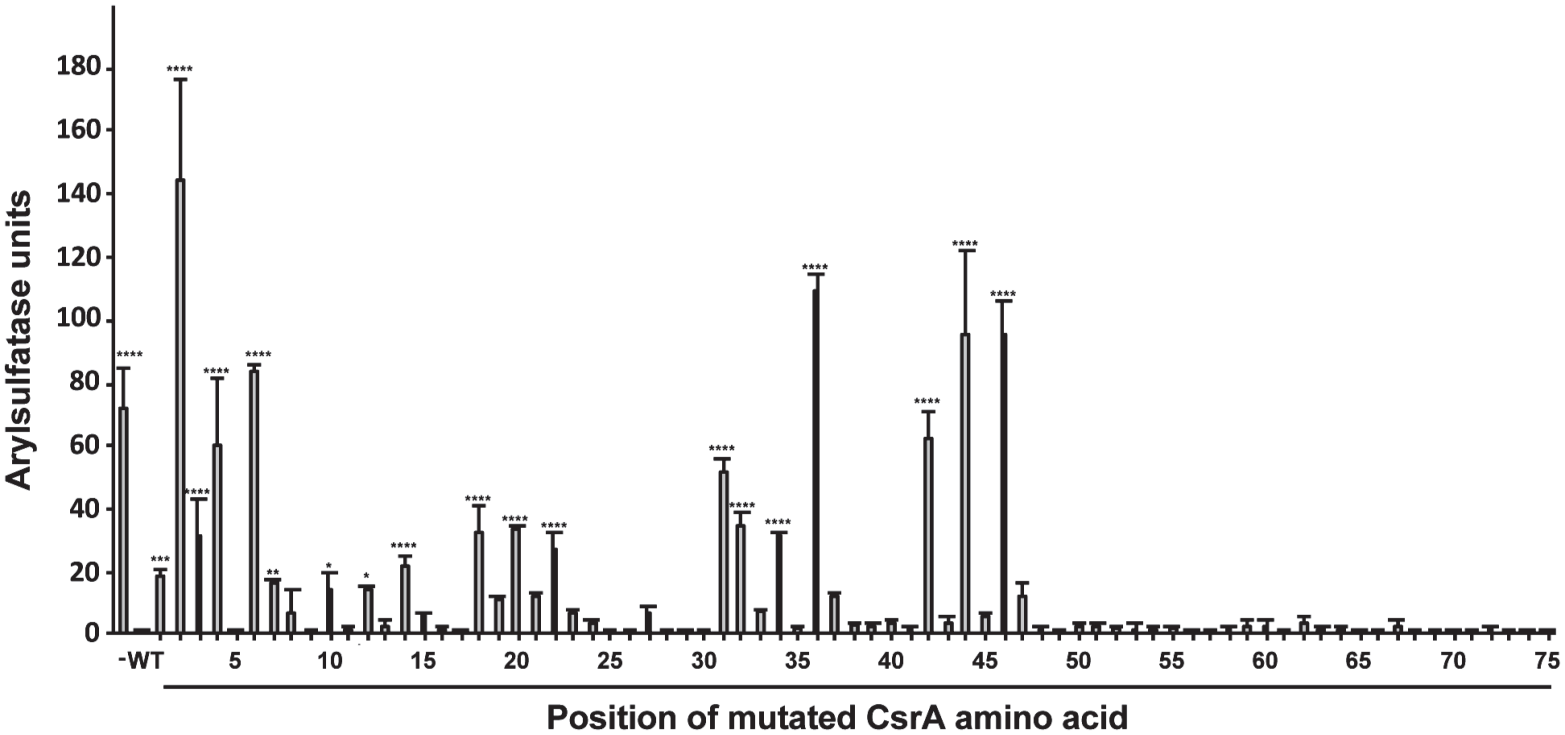
Quantification of regulatory activity by WT and mutant CsrA proteins. *E. coli* BL21(DE3) was co-transformed with the translational reporter pJOFE and either: pET-20b (negative control, labeled “−”), pET-20b-CsrA (positive control, labeled “WT”), or each of the 75 pET-20b-CsrA point mutants. AstA activity in these cells was quantified by arylsulfatase assay (Y axis). The positions of CsrA mutations are indicated below the X axis. Experiments were done a minimum of three times, using triplicate samples. Results were analyzed using one-way ANOVA with Dunnett’s multiple comparisons test, using *p* < 0.05 to indicate significance. ****p < 0.0001, ***p < 0.001, **p < 0.01, *p < 0.05.

To determine the amino acids of CsrA involved in RNA binding, we constructed a translational reporter system. In this system, we cloned DNA encoding the 5′ UTR of *flaA* mRNA upstream of the *C. jejuni* reporter gene *astA*, under the control of the *E. coli lac* promoter (Figure 4). This translational reporter plasmid (pJOFE, Table 1) was co-expressed with the pET-20b alone (negative control), or containing either WT CsrA, or CsrA with 75 individual point mutations. In the absence of CsrA binding to the *flaA* 5′ UTR, AstA activity was high and generated blue colonies (Figure 5, top left, and Figure 6). However, when WT CsrA bound the *flaA* 5′ UTR it greatly repressed AstA expression, resulting in white colonies and low AstA activity (Figure 5, top right, and Figure 6). The colors of colonies expressing CsrA mutants with individual point mutations ranged from light blue to dark blue, indicating qualitatively varying degrees of CsrA activity in binding the *flaA* 5′ UTR (Figure 5, bottom panels, and Supplementary Figure S2).

**Figure 7:**
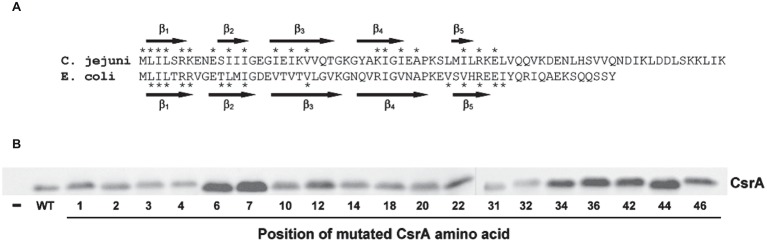
RNA-binding amino acids and expression of CsrA. **(A)** Sequence alignment of *C. jejuni* and *E. coli* CsrA proteins. Proteins were aligned using Clustal Omega (Li et al., 2015), and the location of β strands β_1_-β_5_ were predicted using BETApro (Cheng and Baldi, 2005). Asterisks indicate amino acids involved in RNA binding by the adjacent protein. *E. coli* data were taken from Mercante et al. (2006). **(B)** Expression of *C. jejuni* CsrA proteins in *E. coli* containing pJOFE. *E. coli* cells transformed with the translational reporter pJOFE and pET-20b (“−”), pET-20b-CsrA (“WT”), or the 19 CsrA point mutants that showed significantly higher AstA activity were tested in western blots using CsrA-specific polyclonal antiserum (Fields et al., 2016). All CsrA mutant proteins were expressed at levels equivalent to or higher than WT.

To quantify the degree of CsrA repression of AstA, arylsulfatase assays (Hendrixson and DiRita, 2003) were performed on colonies collected from agar plates (Figure 5). Consistent with plate results, 56 of the 75 site-directed mutants of CsrA exhibited no significant difference in reporter activity compared to WT CsrA (Figure 6). However, CsrA proteins with mutations in 19 amino acids (M1A, L2A, I3A, L4A, R6A, K7A, E10A, I12A, I14A, I18A, I20A, V22A, K31A, I32A, I34A, A36V, I42A, R44A, and E46A) showed significant increases in pJOFE reporter activity, reflecting a decrease in CsrA RNA binding to the *flaA* 5′ UTR (*p* < 0.05) (Figure 6). The amino acid mutations that showed the highest AstA activity were (in decreasing order) L2A, A36V, R44A, E46A, R6A, L4A and I42A (*p* < 0.0001). Most of the detected 19 amino acids were clustered in the five β strands of CsrA predicted by BETApro (Figure 7A; Cheng and Baldi, 2005). We note that some of these CsrA mutations could result in altered CsrA protein structure or potentially non-specific effects on the *E. coli* cells that might affect reporter activity. It was important to exclude the possibility that the site-directed mutants that showed high AstA activity had simply lost CsrA expression, thus we tested the expression of CsrA in the samples used in the arylsulfatase assay by western blot. The expression level of WT CsrA (Figure 7B) was sufficient to give near complete repression of AstA (Figure 6). Although the expression levels of mutant CsrA proteins varied, each of the 19 mutants with high AstA activity had CsrA expression at levels similar to or higher than that of WT (Figure 7B). This indicates that higher reporter activity was not due to poor expression of mutant CsrA proteins.

### Electrophoretic Mobility Shift Assay Shows Decreased RNA Binding by CsrA L2A and A36V

**Figure 8:**
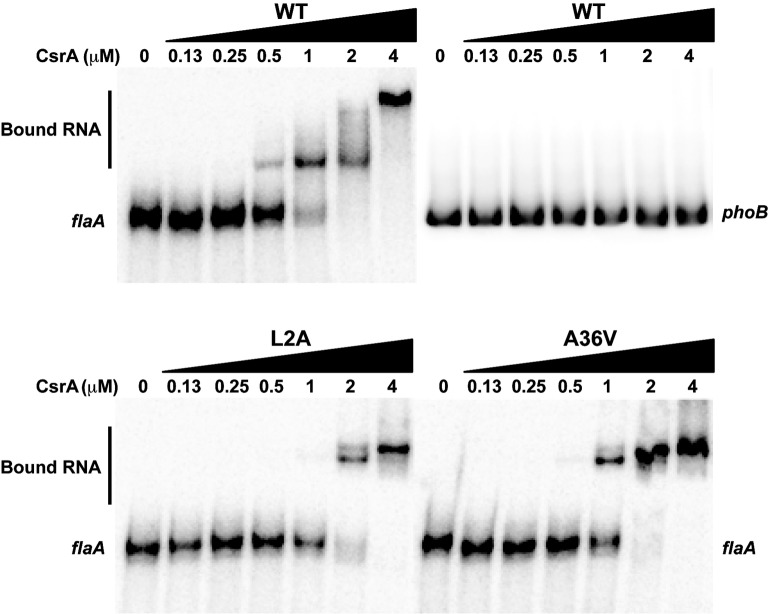
RNA-binding ability of CsrA mutants assessed using EMSA. Purified CsrA-His_6_ proteins (WT, L2A, and A36V) were incubated at different protein concentrations (0–4 μM) with ^32^P-labeled *flaA* mRNA (1 nM), resolved on 12% native polyacrylamide gels, and visualized by a phosphorimager. RNA binding by CsrA resulted in decreased migration of the RNA (“Bound RNA”). Labeled *E. coli phoB* 5′ UTR mRNA was used as a CsrA-non-binding control.

CsrA mutations L2A and A36V showed the most significant loss of CsrA regulatory activity on *flaA* 5′ UTR. To confirm that these CsrA mutants had lost their ability to bind *flaA* mRNA, EMSA was performed using labeled *flaA* 5′ UTR mRNA and different concentrations (0–4 μM) of purified CsrA-His_6_ (WT, L2A or A36V). Labeled *E. coli phoB* 5′ UTR mRNA was used as a CsrA-non-binding control (Patterson-Fortin et al., 2013; Fields et al., 2016). As seen previously (Fields et al., 2016), CsrA WT bound the *flaA* 5′ UTR with shifted species seen at a CsrA concentration as low as of 0.25 μM (Figure 8). Shifts with L2A and A36V occurred only at higher concentrations of the protein, 1 and 0.5 μM, respectively (Figure 8).

### Amino Acids Involved in RNA Binding by CsrA Are Conserved Among *Campylobacter* Species

**Figure 9:**
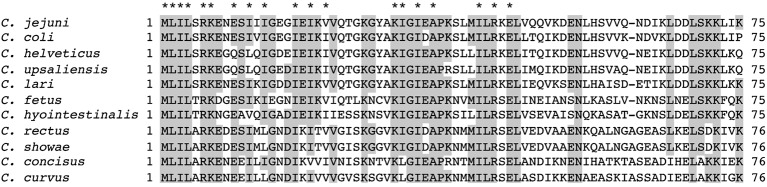
Alignment of CsrA proteins from 11 *Campylobacter* species. CsrA proteins (75 or 76 amino acids in length) from 11 representative *Campylobacter* species were aligned using Clustal Omega (Li et al., 2015). Amino acids that are identical in at least 7 of 11 orthologs are shaded gray. The amino acids identified as having roles in RNA binding by *C. jejuni* CsrA are indicated above the alignment by asterisks and are highly conserved among *Campylobacter* CsrA proteins.

To determine whether the amino acids that were identified as important for the binding of *C. jejuni* CsrA to *flaA* mRNA were conserved among members of the *Campylobacter* genus, we used Clustal Omega (Sievers et al., 2011) to align CsrA proteins from 11 different *Campylobacter* species (Figure 9). Of the 19 CsrA amino acids that had a role in binding *flaA* RNA, 13 were identical among all *Campylobacter* species examined (M1, L2, I3, L4, R6, K7, I18, K31, I34, A36, I42, R44, and E46), with an additional five showing conservative substitutions among the different species (I12, I14, I20, V22, and I32) (Figure 9).

## Discussion

Post-transcriptional control of protein expression by the RNA-binding regulator CsrA is reported in many bacterial species including the gastrointestinal pathogen *C. jejuni* (Fields et al., 2016; Li et al., 2018; Romeo and Babitzke, 2018). CsrA binds target mRNAs and alters their translation or stability (Romeo and Babitzke, 2018). The flagellar protein FlaA is a well-established target of *C. jejuni* CsrA regulation (Dugar et al., 2016; Fields et al., 2016; Radomska et al., 2016). Flagella are considered a major virulence factor in *C. jejuni*, and the extensive transcriptional and post-transcriptional regulation of *C. jejuni* flagellar synthesis ensures proper biosynthesis of flagella (Lertsethtakarn et al., 2011). Furthermore, FlaA is one the most abundant proteins in the cell and flagellar synthesis is energetically costly, so tight regulation of its synthesis is necessary from a metabolic standpoint. The growth-phase dependent regulation of flagellin synthesis (Fields et al., 2016; Li et al., 2018) also suggests that the timing of flagellar assembly could be critical for colonization and pathogenesis. In this study, we used purified *C. jejuni* CsrA to examine CsrA-RNA interactions, using both affinity-selected RNA ligands and *C. jejuni flaA* mRNA as targets.

To begin to understand the mechanisms underlying CsrA-RNA interactions, we first determined high-affinity RNA ligands that are recognized by CsrA. A previous RIP-Seq study identified *C. jejuni* CsrA-binding sites by affinity purification of CsrA-binding RNAs from *C. jejuni* cell lysates, with a consensus sequence of (C/A)A(A/U)GGA found in the loops of stem-loops (Dugar et al., 2016). However, in that study, the presumptive CsrA regulon was composed primarily of FlaA and other flagellar proteins. Because the mRNA encoding FlaA is one of the most abundant transcripts in *C. jejuni* (Dugar et al., 2013), the possibility existed that the CsrA-binding site in that study was heavily influenced by enrichment of transcripts encoding *flaA* and related motility proteins. Since our previous results indicated a much more extensive presumptive CsrA regulon, we chose to use the independent *in vitro* SELEX method for defining the CsrA-binding site. Using SELEX, from a pool of randomized RNA oligonucleotides, we selected an enriched pool of RNA ligands that bind *C. jejuni* CsrA with high affinity (Figure 1). The consensus RNA sequence to which *C. jejuni* CsrA binds is mAwGGAs (Figure 2), and in most cases, the AwGGA motif (Figure 2) was present within the hairpins of stem-loops predicted using MFOLD (Figure 3, Supplementary Figure S1). Importantly, however, our SELEX data also identified atypical CsrA binding sites in which the AwGGA motif is present in the stems of the stem-loops rather than in the loops (Figure 3, Supplementary Figure S1). The importance of binding sites located within stems remains to be determined experimentally, but such sites also occur in mRNAs implicated as CsrA targets in proteome studies of *C. jejuni csrA* and *fliW* mutants (unpublished observations) (Fields et al., 2016; Li et al., 2018). Using both MFOLD analysis and our pJOFE translational reporter, we have performed initial testing of some of the 5′ UTRs upstream of genes encoding putative CsrA targets (Fields et al., 2016; Li et al., 2018) and identified lower affinity targets of CsrA with regulatory sequences resembling the atypical sites identified in our SELEX data (not shown).

The *C. jejuni* CsrA-binding site is similar, but not identical, to the consensus high-affinity RNA-binding site for *E. coli* CsrA, which is RUACARGGAUGU (Dubey et al., 2005). While the nucleotides A_GGA are highly conserved in both species, there is some diversity in the nucleotides surrounding the A_GGA-binding site. SELEX experiments show that the nucleotide immediately preceding the first A in the *C. jejuni* consensus sequence is either C (67%) or A (33%) (ambiguity code “m”). This is somewhat surprising given the low % GC of the *C. jejuni* genome (~30%). Likewise, in 36/37 (97%) of the instances where the AnGGA motif was followed by a C or G (ambiguity code “s”), the C/G nucleotides were present in the predicted loops and not in the adjacent stems. This suggests that these nucleotides were not enriched simply for their abilities to stabilize the stem-loops, but instead may provide specificity to CsrA binding of target mRNAs. The nucleotide immediately preceding the GGA nucleotides is generally A or U (ambiguity code “w”) (84%). The differences in the *C. jejuni* CsrA target sequence compared to that of *E. coli* could in part explain the observation that *C. jejuni* CsrA complements some but not all phenotypes of an *E. coli csrA* mutant (Fields and Thompson, 2012).

Because *C. jejuni* CsrA is rather divergent in amino acid sequence from that of *E. coli* (24% identical/52% similar), our next goal was to determine the amino acids of *C. jejuni* CsrA that are important for RNA binding. To achieve this, we constructed a translational reporter system (pJOFE) in which the *C. jejuni* reporter gene *astA* was cloned downstream of DNA encoding the *flaA* 5′ UTR, under the control of the *E. coli lac* promoter (Figure 4). In the absence of *C. jejuni* CsrA expressed from a compatible vector, *E. coli* cells containing pJOFE appear as large blue colonies (Figure 5, Supplementary Figure S2). When WT CsrA is co-expressed with pJOFE, it binds the *flaA* 5′ UTR and represses the expression of AstA, resulting in small white colonies. It is worth mentioning that *E. coli* colonies with expression of a functional *C. jejuni* CsrA protein are consistently smaller than those not expressing a functional protein, suggesting that *C. jejuni* CsrA is also able to regulate proteins in *E. coli* BL21(DE3) that affect *E. coli* colony size (Figure 5 and data not shown). We next constructed site-directed mutants of each of the 75 amino acids of *C. jejuni* CsrA and tested them for their ability to repress AstA activity from pJOFE, using both qualitative plate and quantitative enzymatic assays. Mutations of CsrA that do not significantly affect CsrA-RNA interaction (56 of 75 mutants in total) give the same results as WT CsrA, appearing on plates as small white colonies, with low AstA enzymatic activity (Figures 5, 6, and data not shown). In contrast, we identified 19 amino acids presumptively involved in CsrA-RNA interaction, yielding large blue colonies similar to the vector control (Figure 5, Supplementary Figure S2). As expected, these mutants all had significantly higher AstA enzymatic activity than WT (Figure 6). Interestingly, the AstA activities of *E. coli* containing the L2A, A36V, R44A, and E46A mutants are somewhat higher than that of cells not expressing *C. jejuni* CsrA. It is possible that these mutants have a non-specific effect on *E. coli* phenotypes related to transcription or translation, as some of these factors are known targets of *E. coli* CsrA (Edwards et al., 2011) and possibly *C. jejuni* CsrA (Fields and Thompson, 2012). These mutants may still bind *flaA* mRNA with reduced affinity compared to WT (Figure 8). However, it is possible that they bind with an altered specificity, for example to the upstream of the two CsrA-binding sites of the *flaA* 5′ UTR (Figure 4) rather than the downstream site that contains the RBS. This could result in stabilization of the mRNA and increased translation. This mechanism of CsrA activation of expression is reported in other bacteria (Patterson-Fortin et al., 2013; Yakhnin et al., 2013; Ren et al., 2014; Romeo and Babitzke, 2018).

Of the 19 identified amino acids, 11 were at positions previously identified as important for the regulatory activity of *E. coli* CsrA (Mercante et al., 2006). These amino acids tended to cluster within the five predicted β strands of CsrA, with the most significant amino acids present in or near the β_1_ and β_5_ strands (Figure 7A). In known structures of CsrA orthologs, these two β strands form an edge of inter-subunit β-sheet (Gutierrez et al., 2005; Rife et al., 2005; Heeb et al., 2006), where CsrA binds its target mRNA (Schubert et al., 2007). In *C. jejuni* CsrA, L2A shows the greatest loss in regulatory activity based on results from both arylsulfatase assay and EMSA gel shifts, followed by A36V, R44A, E46A, R6A, L4A, and I42A. This is somewhat different than in *E. coli*, in which the CsrA mutants that had the strongest RNA-binding phenotypes were (in decreasing order) R44A, V42A, L2A, I47A, V40A, L4A, R6A, and R7A (Mercante et al., 2006). While *C. jejuni* CsrA mutant I42A shows significantly reduced regulatory activity, the phenotype is not as strong as the analogous mutation in *E. coli* CsrA. Amino acid R44 is a significant residue for CsrA-RNA interaction in *Yersinia enterocolitica* (Heeb et al., 2006), while in *Pseudomonas fluorescens* mutation of R44 and L4 causes loss of RsmE (CsrA) ability to repress its target mRNA (Schubert et al., 2007). While the reduced regulatory activity of the *C. jejuni* CsrA mutants is likely due to the importance of the mutated amino acids in RNA interactions, it is also possible that some of the mutations affect overall CsrA protein structure, although the use of alanine as the substituted amino acid is a standard approach to minimize such disruptions. The secondary structure of the CsrA mutant proteins was predicted using two different programs [BETApro and PredictProtein (not shown)], and β strands were present in all of the mutant proteins. However, the two programs made slightly different predictions, with some subtle variations in β strand locations. Thus, without an experimentally determined structure of CsrA, predicted secondary structures of the mutants cannot be confirmed. Furthermore, we cannot exclude potential non-specific effects of the mutations on *E. coli* as described above.

The nuclear magnetic resonance (NMR) structure of CsrA ortholog from *P. fluorescens* (RsmE) complexed to a target mRNA indicates that RNA-binding surfaces are highly positively charged and formed by the aforementioned edges of β-sheets composed of the β_1_ and β_5_ strands of the opposite subunits of the dimer and the regions around the β_3_-β_4_ and β_4_-β_5_ loops (Schubert et al., 2007). The GG dinucleotide within the consensus RNA-binding sequence (A/U) CANGGANG (U/A) is located toward the hydrophobic core, close to L2 and L4 of β_1_ of one subunit and V42 of β_5_ of the opposite subunit. This dinucleotide is specifically recognized *via* interactions with the protein backbone within the β_5_ strand and the β_4_–β_5_ loop (Schubert et al., 2007). Electrostatic contacts between RNA and CsrA R44 are crucial for the formation of a stable complex (Schubert et al., 2007). The presence of a salt bridge between R6 and E46 is indispensable to maintain structure and biological activity of RsmE (Heeb et al., 2006; Schubert et al., 2007). Interestingly, structural data indicate that the specificity of RNA recognition by CsrA is primarily a product of interactions of target RNA nucleotides with the protein backbone rather than the amino acid side chains (Schubert et al., 2007; Morris et al., 2013). Future structural studies are warranted to determine how mutations in *C. jejuni* CsrA affect the overall structure of the protein and its RNA-binding properties. However, our results are consistent with amino acids and regions previously identified in other CsrA orthologs playing a role in RNA binding by *C. jejuni* CsrA.

To exclude the possibility that the mutants with reduced regulatory activity had lost CsrA expression, we performed western blots on the same samples used in the arylsulfatase assays and showed that each of the 19 CsrA mutants has expression levels similar to or higher than that of WT CsrA (Figure 7B). This confirms that the reduced regulatory activity of these mutants was due specifically to loss of protein functionality rather than poor CsrA expression. To confirm that reduced CsrA regulatory activity was due to altered RNA binding, we performed EMSA using purified proteins of the two most significant mutants (L2A and A36V) and radiolabeled *flaA* mRNA. These experiments showed decreased RNA binding by both mutants relative to WT (Figure 8), as shifts occurred only at higher concentrations of CsrA. The CsrA amino acids of *C. jejuni* detected in this study as being important for CsrA regulatory activity on *flaA* mRNA are highly conserved among 11 selected *Campylobacter* species, with 13 of the 19 amino acids being identical and five being conservative substitutions (Figure 9). Nine of the 19 identified amino acids (L2, R6, K7, I14, I18, A36, I42, R44, and E46) are also conserved in CsrA proteins from diverse bacterial species (Fields and Thompson, 2012).

Identification of the consensus CsrA-binding site and amino acids critical for CsrA binding to *flaA* mRNA serves as a model for studying *C. jejuni* CsrA interaction with other important target mRNAs. The findings of this study are also a precursor to fully understand the mechanism of antagonism of *C. jejuni* CsrA by the flagellar chaperone FliW. In *B. subtilis,* FliW inhibits CsrA RNA binding by a noncompetitive allosteric mechanism where FliW binds CsrA at a surface distinct from its RNA-binding pocket (Mukherjee et al., 2016). Ongoing studies by our group are exploring whether FliW antagonizes CsrA activity toward target mRNAs through direct competition for the CsrA RNA-binding site, by steric hindrance, or by a noncompetitive allosteric mechanism. In addition, understanding the mechanism by which CsrA regulates the expression of a major *C. jejuni* virulence factor (flagella) may allow the development of novel strategies to limit *C. jejuni* infection.

## Data Availability

The datasets generated for this study are available on request to the corresponding author.

## Author Contributions

FE, JL, HO, CD, CF, MB, and ST contributed to the conception and design of the study. FE performed the statistical analysis and wrote the first draft of the manuscript. FE, MB, and ST wrote sections of the manuscript. All authors contributed to manuscript revision, read and approved the submitted version.

### Conflict of Interest Statement

The authors declare that the research was conducted in the absence of any commercial or financial relationships that could be construed as a potential conflict of interest.
