# Supplementary material for: RNA Binding by the Campylobacter jejuni Post-transcriptional Regulator CsrA
Source: Front Microbiol. 2019 Aug 7;10:1776. doi: 10.3389/fmicb.2019.01776 (PMC6692469; doi:10.3389/fmicb.2019.01776)
Supplement: Supplementary file 1 [file Data_Sheet_1.pdf]

Supplementary Table S1. PCR primers used in this study

SELEX PCR primers

Primer	Sequence (5′ -> 3′)	Reference
SELEX15	ACCGAGTCCAGAAGCTTGTAGTAC(N15)GCCTAGATGG AGTTGAATTCTCCCTATAGTGAGTCGTATTAC	(Tuerk and Gold 1990)
P1	GTAATACGACTCACTATAGGGAGAATTCAACTCCATCTA	(Tuerk and Gold 1990)
P2	ACCGAGTCCAGAAGCTTGTAGT	(Tuerk and Gold 1990)

SDM and Translational reporter PCR primers

Primer	Sequence (5′ -> 3′)	Description
**Forward**		
csrA.M1A.F2	CATATG**GC**GTTAATATTATCAAGAAAAGAAAATGA	M1A
csrA.L2A.F2	CATATG**GC**AATATTATCAAGAAAAGAAAATGA	L2A
csrA.I3A.F2	CATATGTTA**GC**ATTATCAAGAAAAGAAAATGA	I3A
csrA.L4A.F2	CATATGTTAATA**GC**ATCAAGAAAAGAAAATGA	L4A
csrA.S5A.F2	CATATGTTAATATTA**GC**AAGAAAAGAAAATGA	S5A
csrA.R6A.F2	CATATGTTAATATTATCA**GC**AAAAGAAAATGA	R6A
csrA.K7A.F2	CATATGTTAATATTATCAAGA**GC**AGAAAATGAAAGTAT	K7A
csrA.E8A.F2	CATATGTTAATATTATCAAGAAAA**GC**AAATGAAAGTAT	E8A
csrA.N9A.F2	CATATGTTAATATTATCAAGAAAAGAA**GC**TGAAAGTAT	N9A
csrA.E10A.F2	CATATGTTAATATTATCAAGAAAAGAAAAT**GC**AAGTAT	E10A
csrA.S11A.F	A**GC**TATAATTATCGGAGAAGGTATAG	S11A
csrA.I12A.F	AAGT**GC**AATTATCGGAGAAGGTATAG	I12A
csrA.I13A.F	AAGTATA**GC**TATCGGAGAAGGTATAG	I13A
csrA.I14A.F	AAGTATAATT**GC**CGGAGAAGGTATAG	I14A
csrA.G15A.F	CG**C**AGAAGGTATAGAAATCAAAG	G15A
csrA.E16A.F	CGGAG**C**AGGTATAGAAATCAAAG	E16A
csrA.G17A.F	CGGAGAAG**C**TATAGAAATCAAAG	G17A
csrA.I18A.F	CGGAGAAGGT**GC**AGAAATCAAAG	I18A
csrA.E19A.F	G**C**AATCAAAGTCGTTCAAACAGG	E19A
csrA.I20A.F	GAA**GC**CAAAGTCGTTCAAACAGG	I20A
csrA.K21A.F	GAAATC**GC**AGTCGTTCAAACAGG	K21A
csrA.V22A.F	GAAATCAAAG**C**CGTTCAAACAGG	V22A
csrA.V23A.F	G**C**TCAAACAGGGAAAGGATATGC	V23A
csrA.Q24A.F	GTT**GC**AACAGGGAAAGGATATGC	Q24A
csrA.T25A.F	GTTCAA**G**CAGGGAAAGGATATGC	T25A
csrA.G26A.F	GTTCAAACAG**C**GAAAGGATATGC	G26A
csrA.K27A.F	**GC**AGGATAcGCaAAAATAGGaATAGAAGC	K27A
csrA.G28A.F	ACAGGGAAAG**CT**TATGCCAAAATAG	G28A
csrA.Y29A.F	AGGGAAAGGA**GC**TGCCAAAATAGG	Y29A
csrA.A30V.F	AAAGGATATG**T**CAAAATAGGCATAG	A30V
csrA.K31A.F	C**GC**AATAGGaATAGAAGCTCCAAAATC	K31A
csrA.I32A.F	CAAA**GC**AGGCATAGAAGCTCCAAAATC	I32A
csrA.G33A.F	CAAAATAG**C**CATAGAAGCTCCAAAATC	G33A
csrA.I34A.F	CAAAATAGGC**GC**AGAAGCTCCAAAATC	I34A
csrA.E35A.F	AG**C**AGCTCCAAAATCCCTTATGATAC	E35A
csrA.A36V.F	AGAAG**T**TCCAAAATCCCTTATGATAC	A36V
csrA.P37A.F	AGAAGCT**G**CAAAATCCCTTATGATAC	P37A
csrA.K38A.F	AGAAGCTCCA**GC**ATCCCTTATGATACTTAGAAAA	K38A
csrA.S39A.F	AGCTCCAAAg**G**CCCTTATGATAC	S39A
csrA.L40A.F	TCC**GC**TATGATACTTAGAAAA	L40A
csrA.M41A.F	TCCCTT**GC**GATACTTAGAAAA	M41A
csrA.I42A.F	TCCCTTATG**GC**ACTTAGAAAA	I42A
csrA.L43A.F	GATA**GC**TAGAAAAGAACTTGTTCAACAAG	L43A
csrA.R44A.F	GATACTT**GC**AAAAGAACTTGTTCAACAAG	R44A
csrA.K45A.F	GATACTTAGA**GC**AGAACTTGTTCAACAAG	K45A
csrA.E46A.F	AG**C**ACTTGTTCAACAAGTAAAGGATG	E46A
csrA.L47A.F	AGAA**GC**TGTTCAACAAGTAAAGGATG	L47A
csrA.V48A.F	AGAACTTG**C**TCAACAAGTAAAGGATG	V48A

csrA.Q49A.F	AGAACTTGTT**GC**ACAAGTAAAGGATG	Q49A
csrA.Q50A.F	TCAA**GC**AGTAAAGGATGAAAATTTGCATTCTGTTG	Q50A
csrA.V51A.F	TCAACAAG**C**AAAGGATGAAAATTTGCATTCTGTTG	V51A
csrA.K52A.F	TCAACAAGTA**GC**GGATGAAAATTTGCATTCTGTTG	K52A
csrA.D53A.F	GG**C**TGAAAATTTGCATTCTGTTGTTCAGAATGATATTAAAC	D53A
csrA.E54A.F	GGATG**C**AAATTTGCATTCTGTTGTTCAGAATGATATTAAAC	E54A
csrA.N55A.F	GGATGAA**GC**TTTGCATTCTGTTGTTCAGAATGATATTAAAC	N55A
csrA.L56A.F	GGATGAAAAT**GC**GCATTCTGTTGTTCAGAATGATATTAAAC	L56A
csrA.H57A.F	G**GC**TTCTGTTGTTCAGAATGATATTAAAC	H57A
csrA.S58A.F	GCAT**G**CTGTTGTTCAGAATGATATTAAAC	S58A
csrA.V59A.F	GCATTCTG**C**TGTTCAGAATGATATTAAAC	V59A
csrA.V60A.F	GCATTCTGTTG**C**TCAGAATGATATTAAAC	V60A
csrA.Q61A.F	T**GC**GAATGATATTAAACTTGATGACTTAAG	Q61A
csrA.N62A.F	TCAG**GC**TGATATTAAACTTGATGACTTAAG	N62A
csrA.D63A.F	TCAGAATG**C**TATTAAACTTGATGACTTAAG	D63A
csrA.I64A.F	TCAGAATGAT**GC**TAAACTTGATGACTTAAG	I64A
csrA.K65A.F	ATT**GC**ACTTGATGACTTAAGCAAAAAAC	K65A
csrA.L66A.F	ATTAAA**GC**TGATGACTTAAGCAAAAAAC	L66A
csrA.D67A.F	ATTAAACTTG**C**TGACTTAAGtAAAAAAC	D67A
csrA.68-75.F	CACCACCACCACCACCACTGAGAT	F for mutants 68-75
FME01	GCGCATATGTTAGACCTTTCTCTTCAATAAG	NdeI site
FME03	AAACATATGAGACTTAGCAAAACTCTTTGTATGGC	NdeI site
JO-4	AAAGCTAGCGTCGACTGGCCGATTCATTAATGCAGCTGGC	NheI/SalI sites

Reverse

csrA.1-10.R2	TATATCTCCTTCTTAAAGTTAAACAAAATTATTT	R mutants 1-10
csrA.11-14.R	TCATTTTCTTTTCTTGATAATATTAAC	R for mutants 11-14
csrA.15-18.R	ATAATTATACTTTCATTTTCTTTTCTTG	R for mutants 15-18
csrA.19-22.R	TATACCTTCTCCGATAATTATAC	R for mutants 19-22
csrA.23-26.R	GACTTTGATTTCTATACCTTC	R for mutants 23-26
csrA.27&30.R	CCCTGTTTGAACGACTTTG	R for mutants 27&30
csrA.28.R	TTGAACGACTTTGATTTCTATAC	R for mutant 28
csrA.29.R	GTTTGAACGACTTTGATTTC	R for mutant 29
csrA.31-34.R	GCATATCCTTTCCCTGTTTG	R for mutants 31-34
csrA.35-38.R	ATtCCTATTTTGGCATATC	R for mutants 35-38
csrA.39.R	TCTATGCCTATTTTtGCgTATCCTTTCCC	R for mutant 39
csrA.40-42.R	TTTTGGAGCTTCTATGCCTAT	R for mutants 40-42
csrA.43-45.R	ATAAGGGATTTTGGAGCTTCTATGC	R for mutants 43-45
csrA.46-49.R	TTTCTAAGTATCATAAGGGATTTTGGAGC	R for mutants 46-49
csrA.50-52.R	ACAAGTTCTTTTCTAAGTATCATAAG	R for mutants 50-52
csrA.53-56.R	TTTACTTGTTGAACAAGTTCTTTTC	R for mutants 53-56
csrA.57-60.R	AAATTTTCATCCTTTACTTGTTGAAC	R for mutants 57-60
csrA.61-64.R	ACAACAGAATGCAAgTTTTCATCCTT	R for mutants 61-64
csrA.65-67.R	ATCgTTtTGAACAACAGAATG	R for mutants 65-67
csrA.D68A.R2	CTCGAGTTTGATTAGTTTTTTGCTTAAG**G**CATCAAG	D68A
csrA.L69A.R2	CTCGAGTTTGATTAGTTTTTTGCTT**GC**GTCATCAAG	L69A
csrA.S70A.R2	CTCGAGTTTGATTAGTTTTTTG**GC**TAAGTCATCAAG	S70A
csrA.K71A.R2	CTCGAGTTTGATTAGTTTT**GC**GCTTAAGTC	K71A
csrA.K72A.R2	CTCGAGTTTGATTAGT**GC**TTTGCTTAAGTC	K72A
csrA.L73A.R2	CTCGAGTTTGATT**GC**TTTTTTGCTTAAGTC	L73A
csrA.I74A.R2	CTCGAGTTTG**GC**TAGTTTTTTGCTTAAGTC	I74A
csrA.K75A.R2	CTCGAGT**GC**GATTAGTTTTTTGCTTAAGTC	K75A
FME02	CCCCATATGCATTTTAAATCCTTTTAAATAATTTCAAAC	NdeI site
FME04	AAAGCGGCCGCGTCGACAAACAAATTTACAAAAAA	NotI/SalI sites
JO-5	AAAGCTAGCGGAGAGGCGGTTTGCGTATTGGG	NheI site

Nucleotides that were altered to substitute amino acids are indicated in bold and underlined. Nucleotides indicated in lower case were changed to eliminate secondary structure in the primer, but without changing the encoded amino acid. Restriction sites for cloning purposes are indicated with single or double underlines.

Supplementary Figure S1. MFOLD structures of SELEX products

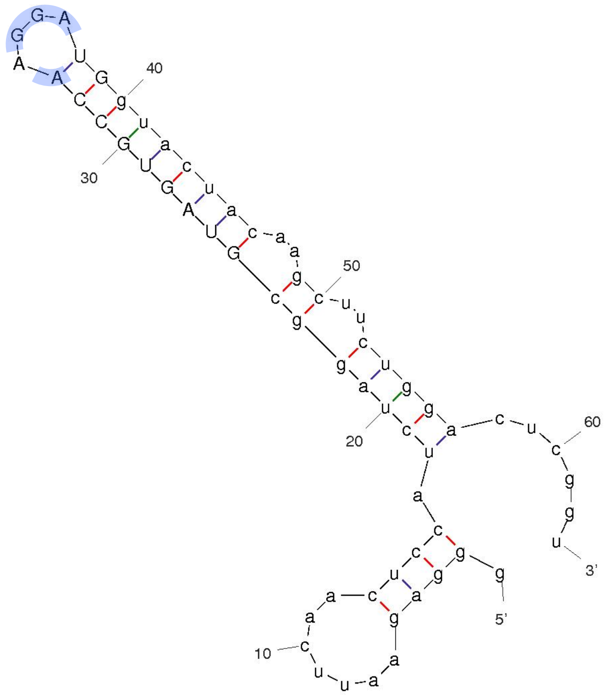

10-1

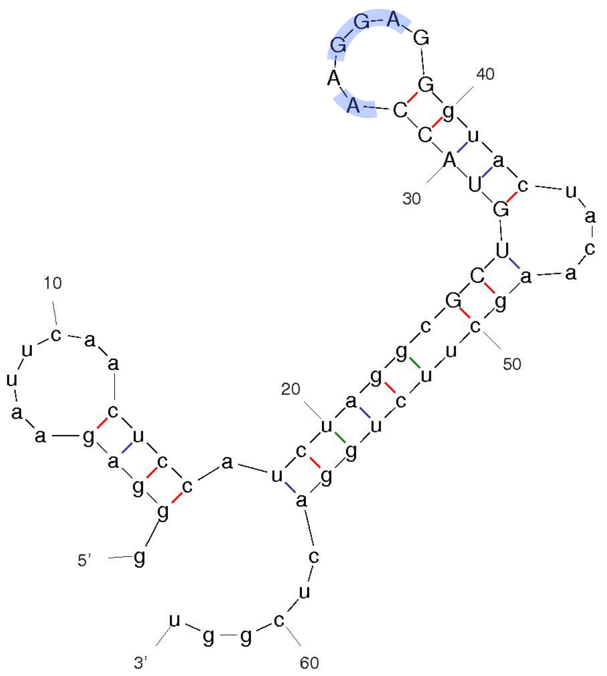

9-11

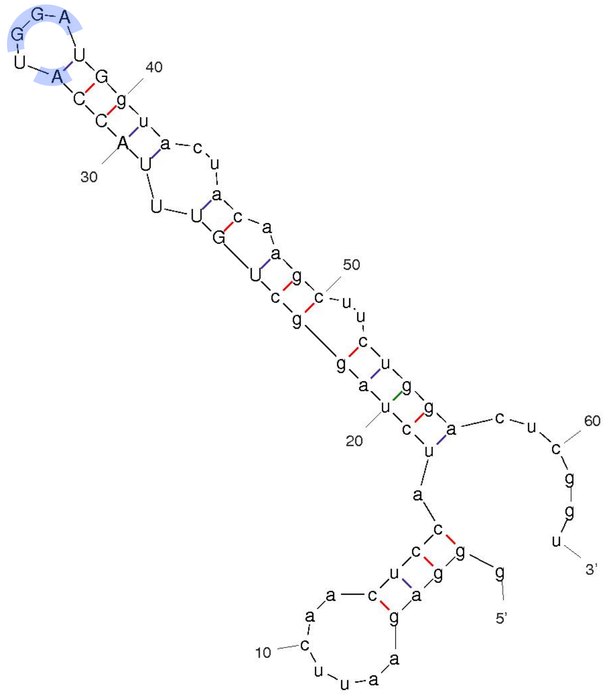

9-17

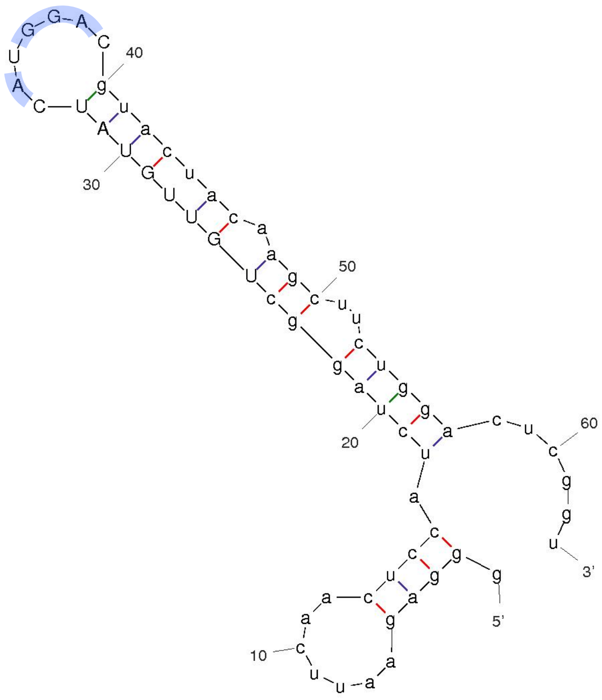

10-8

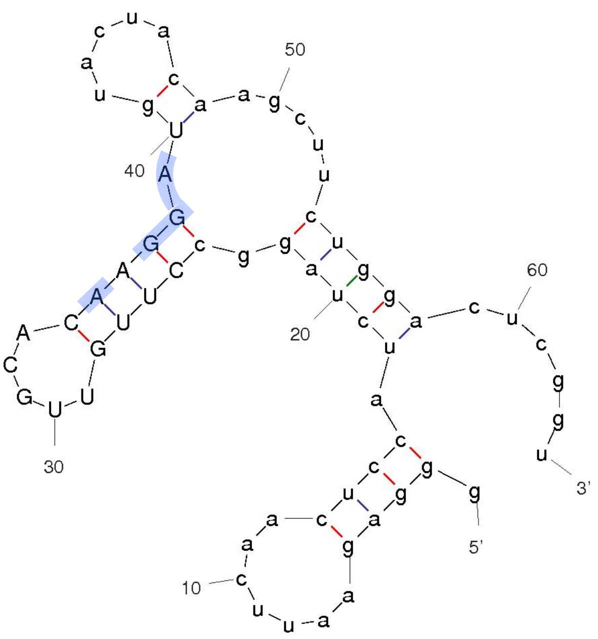

10-6

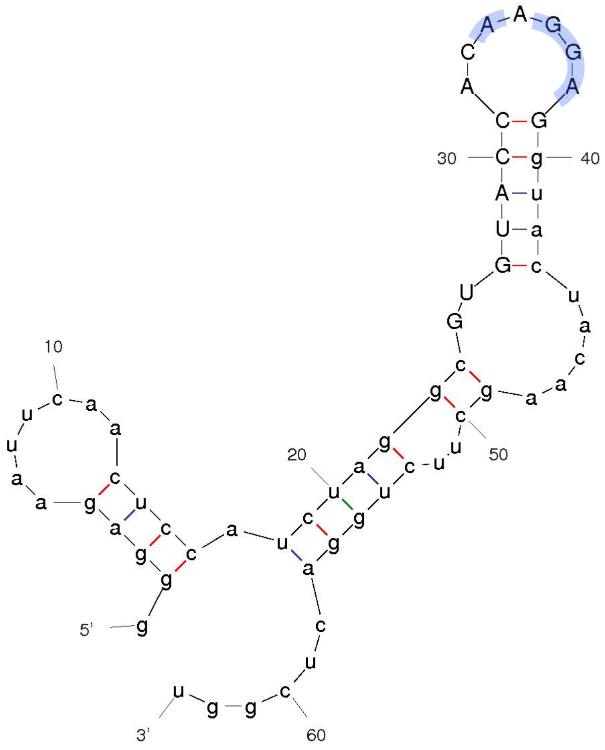

9-27

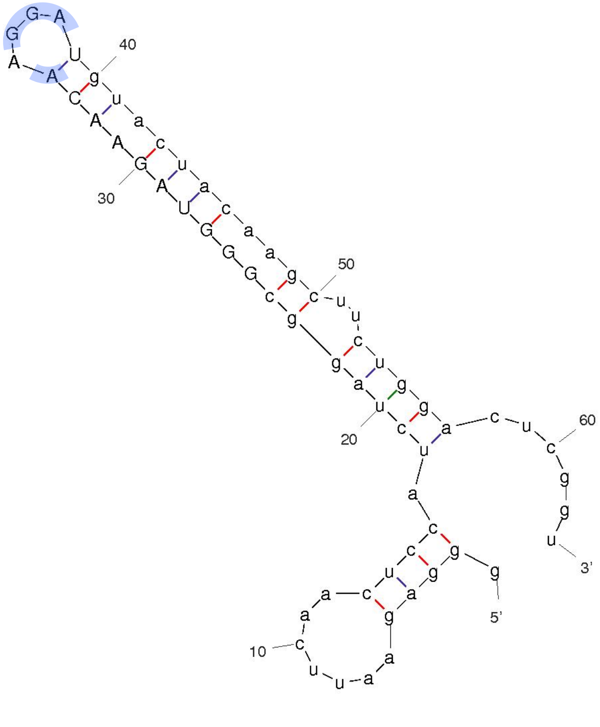

9-32

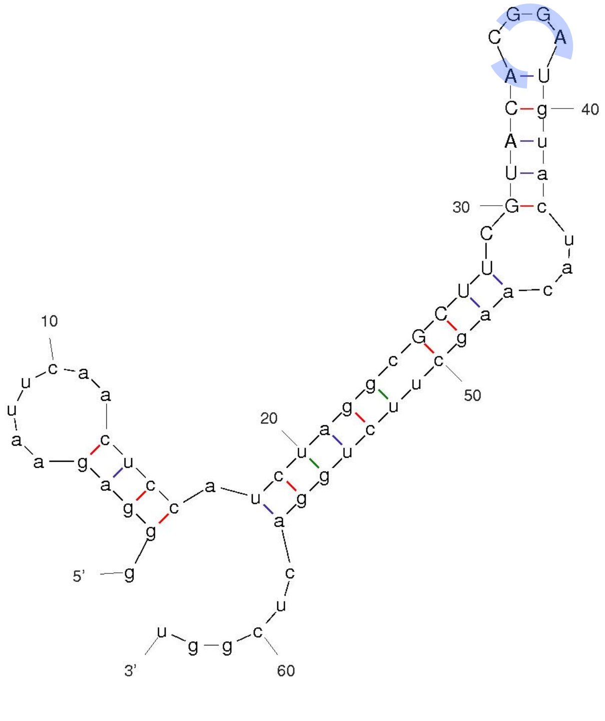

10-3

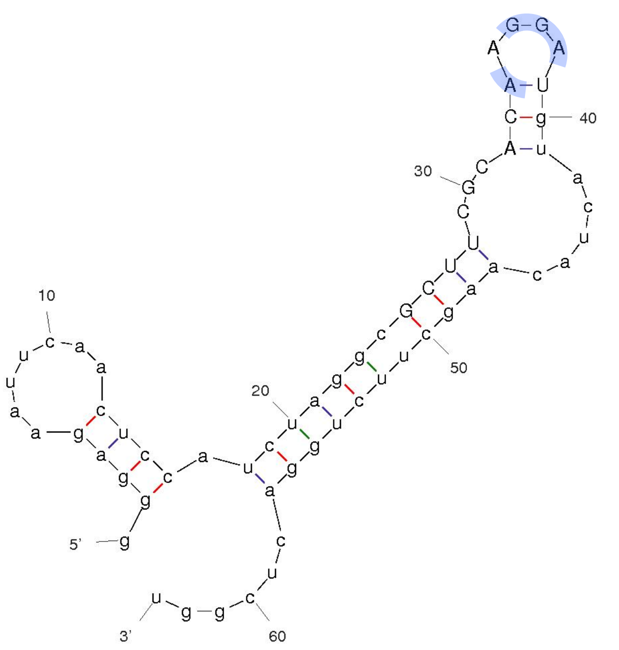

10-10

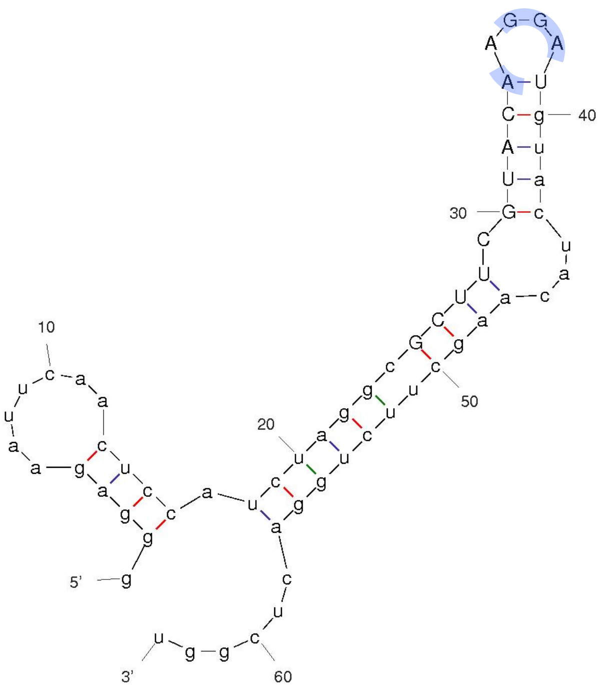

10-15

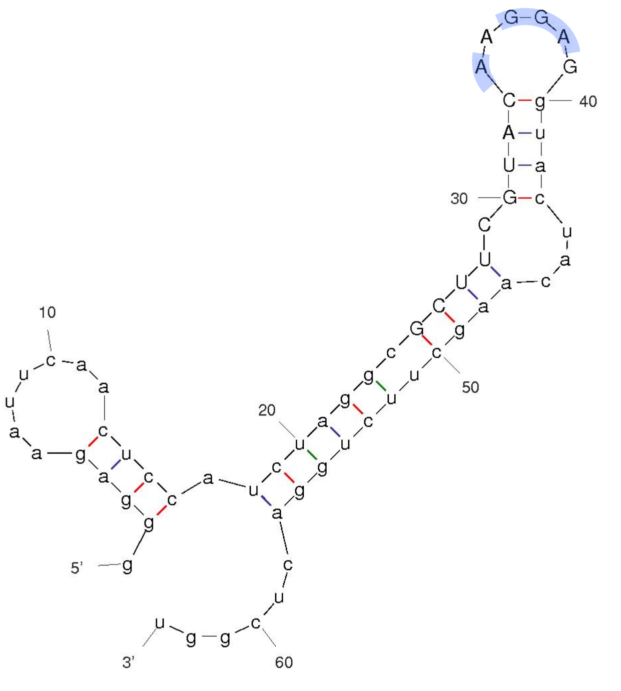

9-7

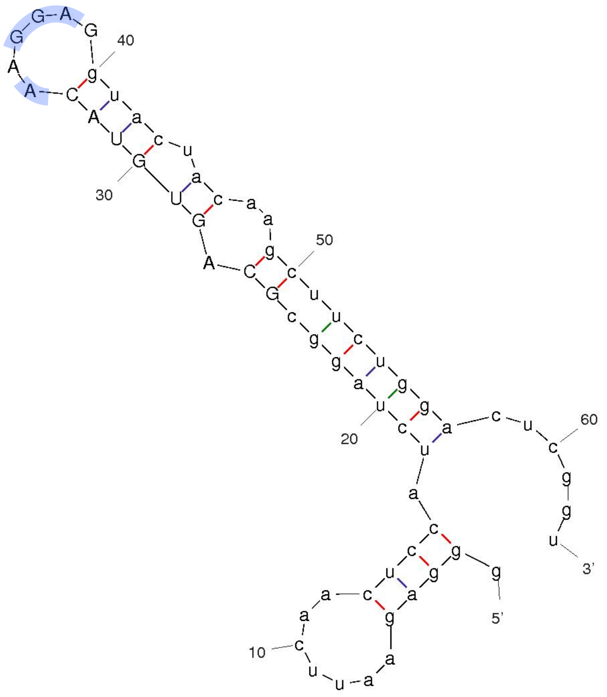

10-17

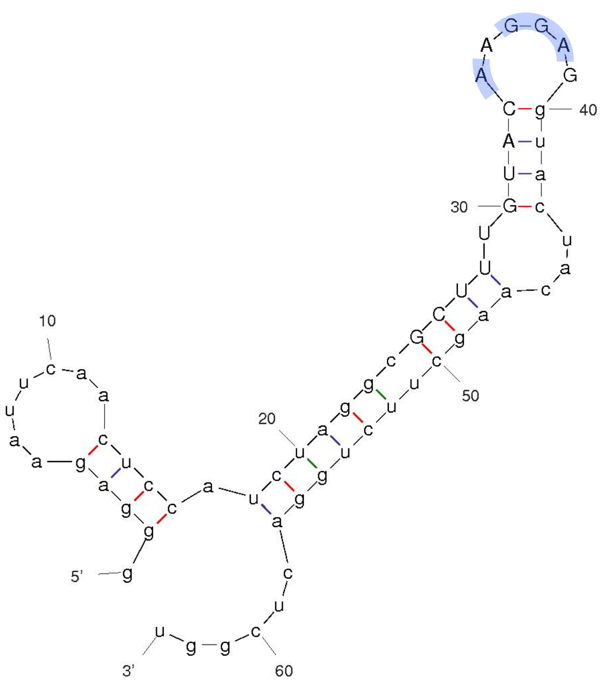

9-38

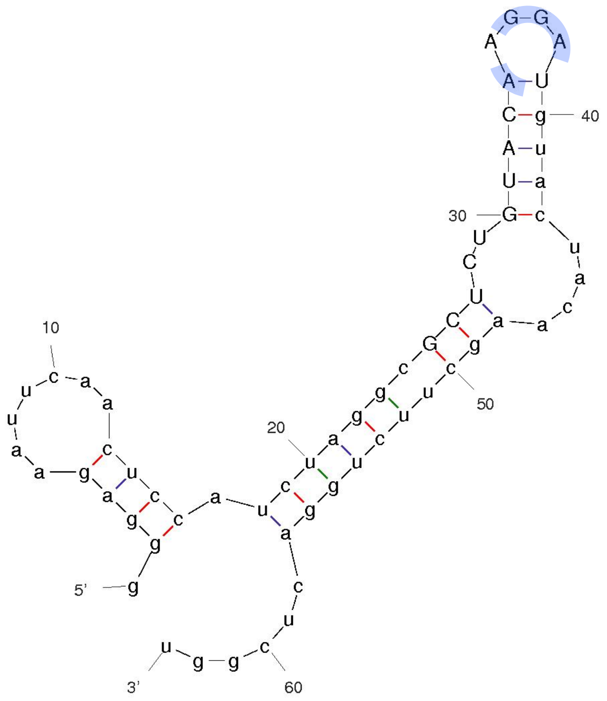

10-34

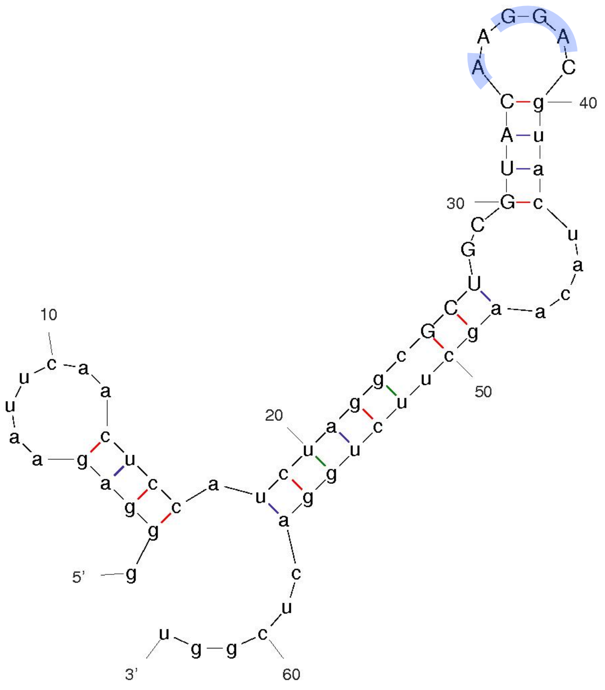

9-36

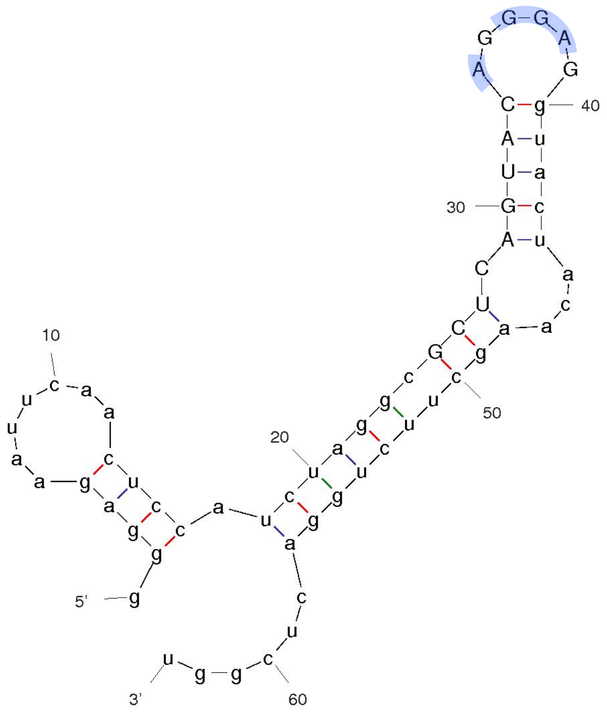

10-19

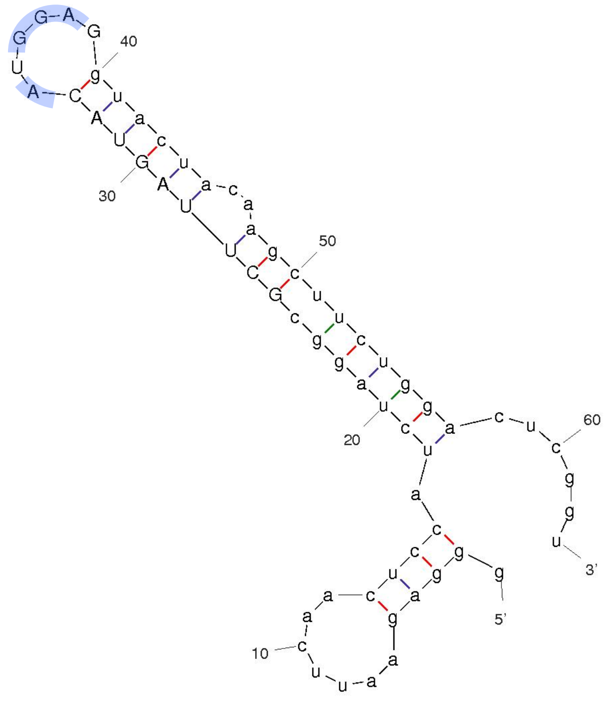

10-30

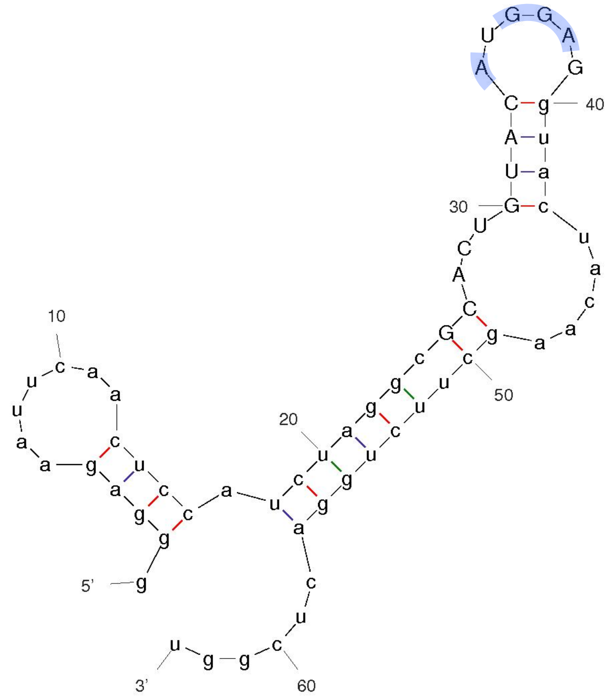

10-25

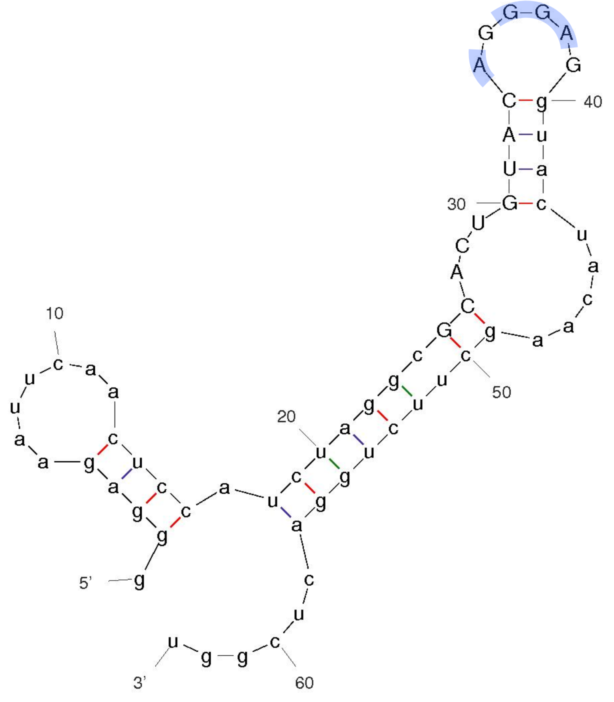

10-32

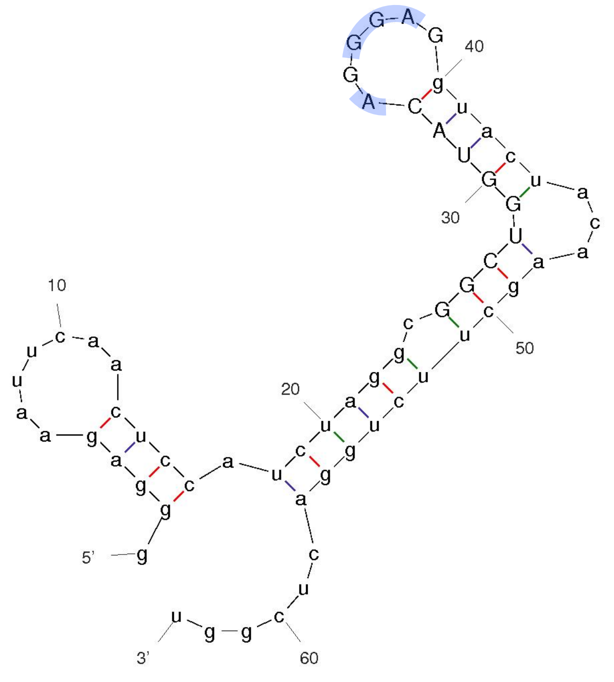

9-15

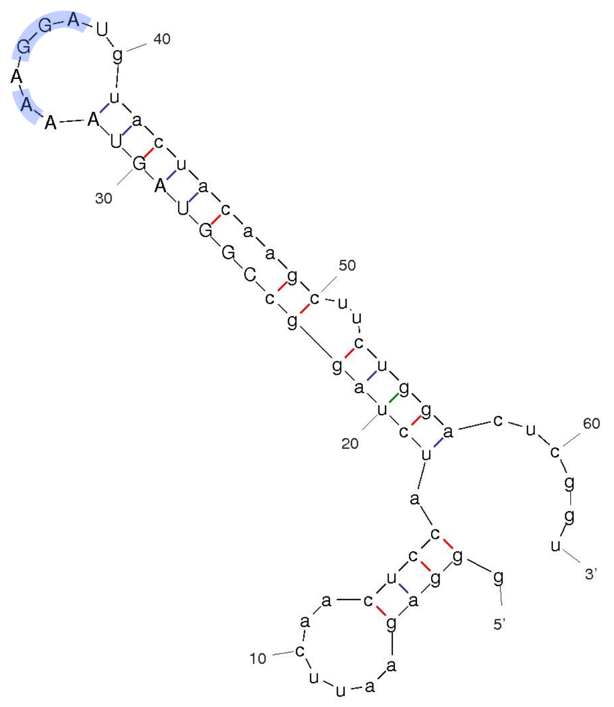

10-39

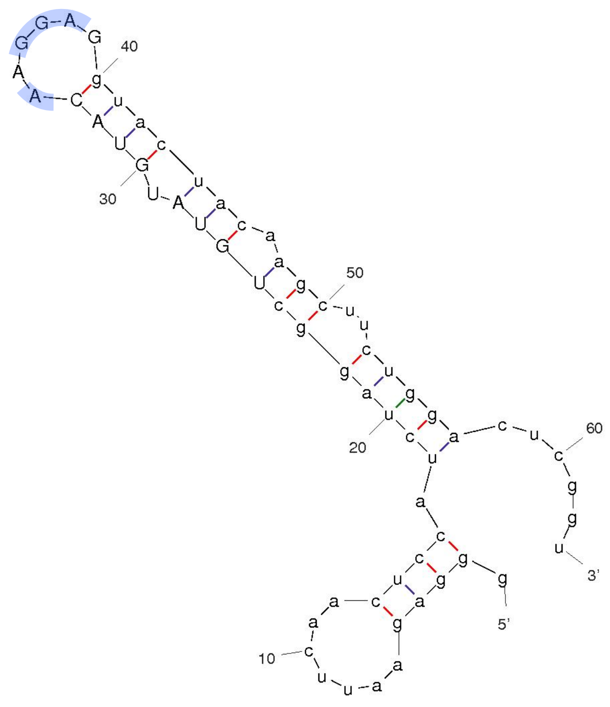

9-12

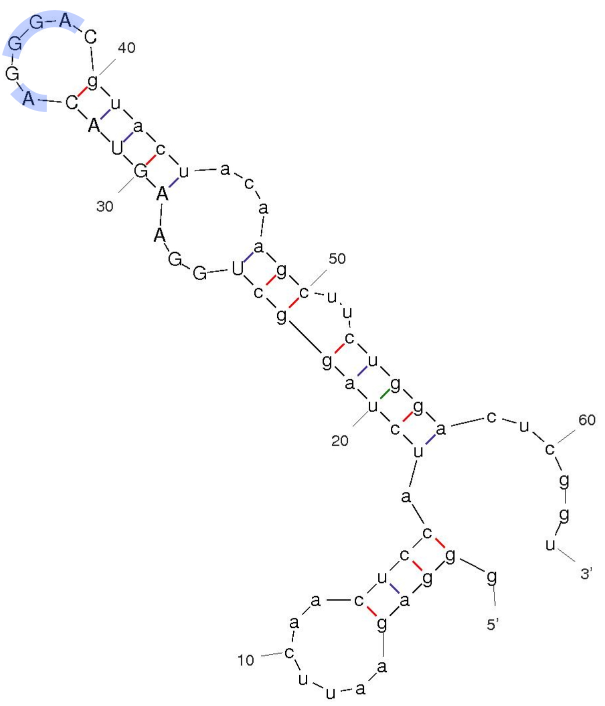

9-35

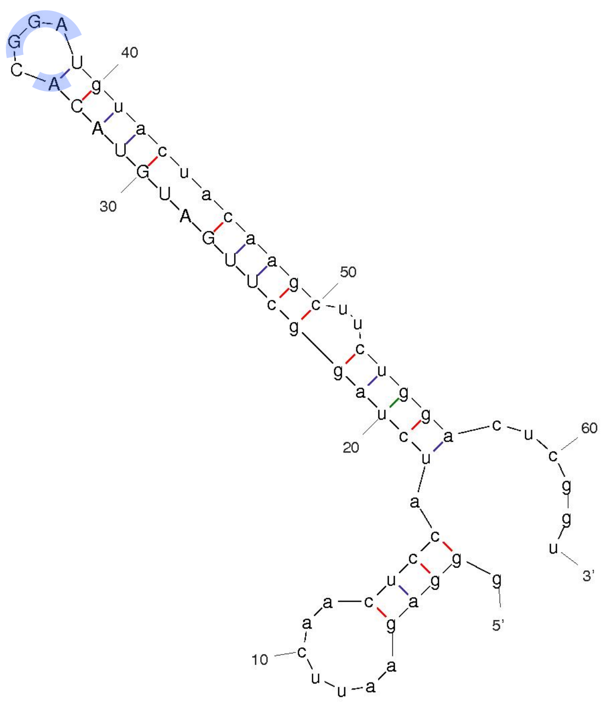

10-18

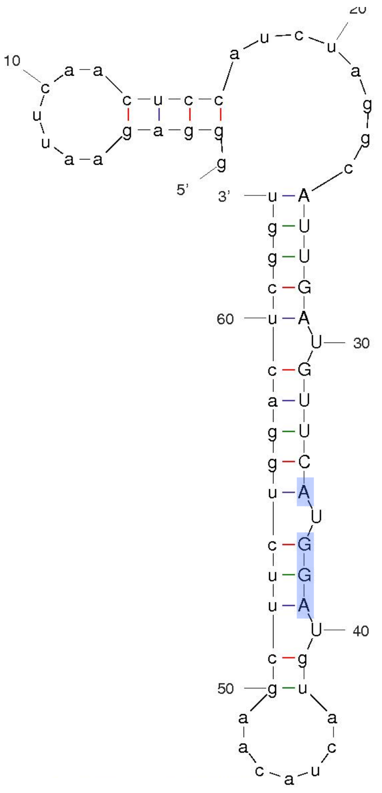

9-13

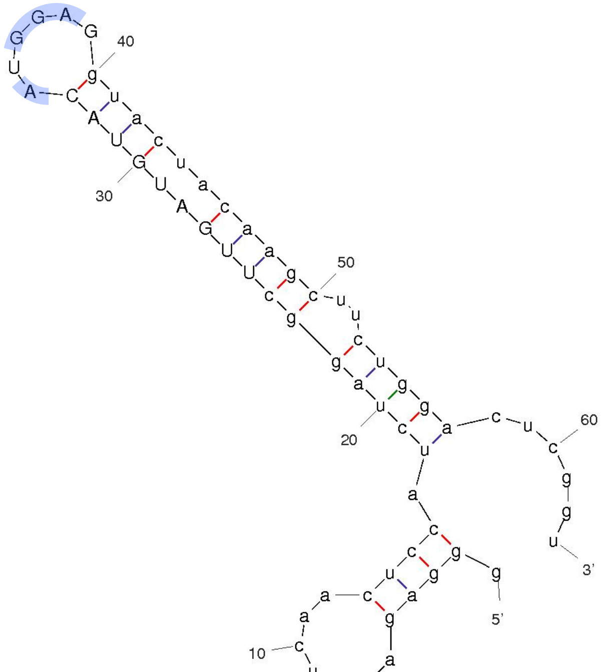

10-9

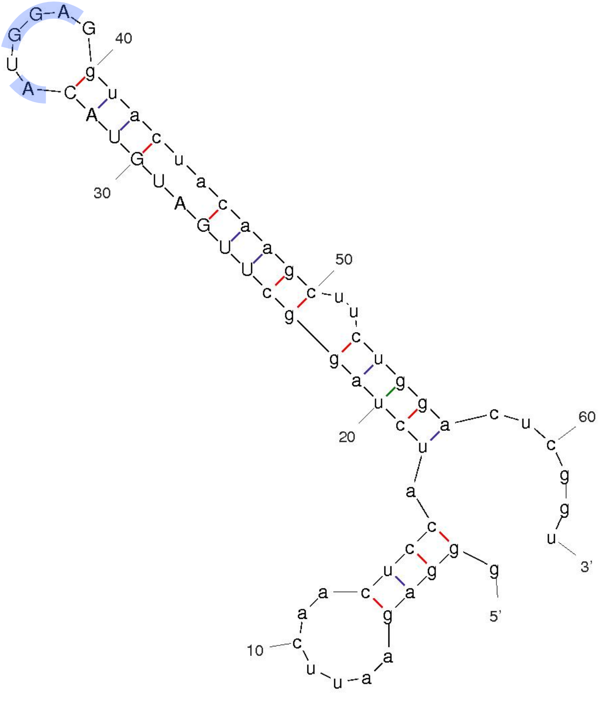

10-7

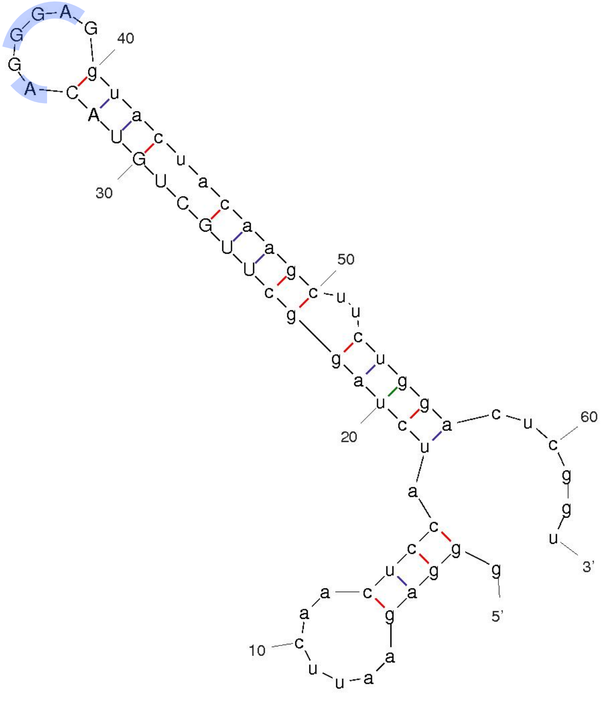

9-40

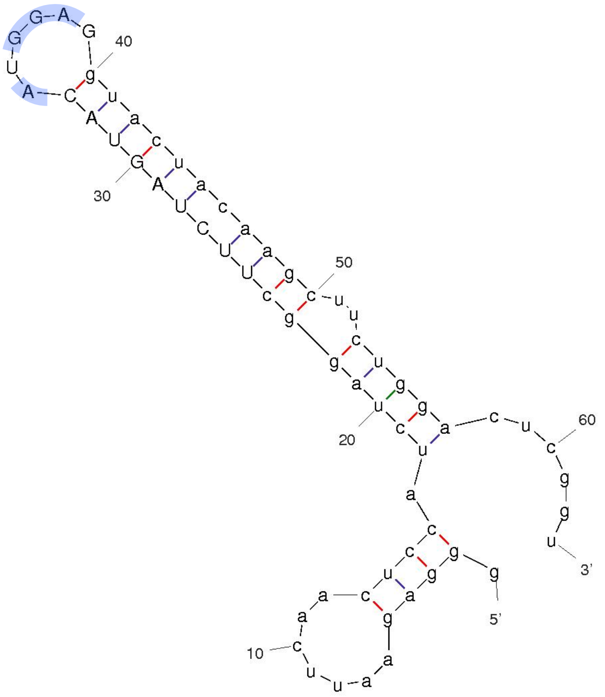

10-13

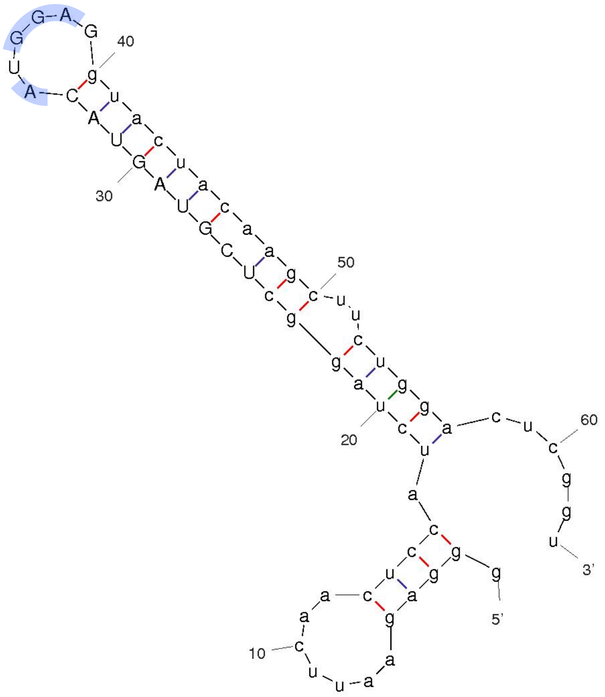

10-33

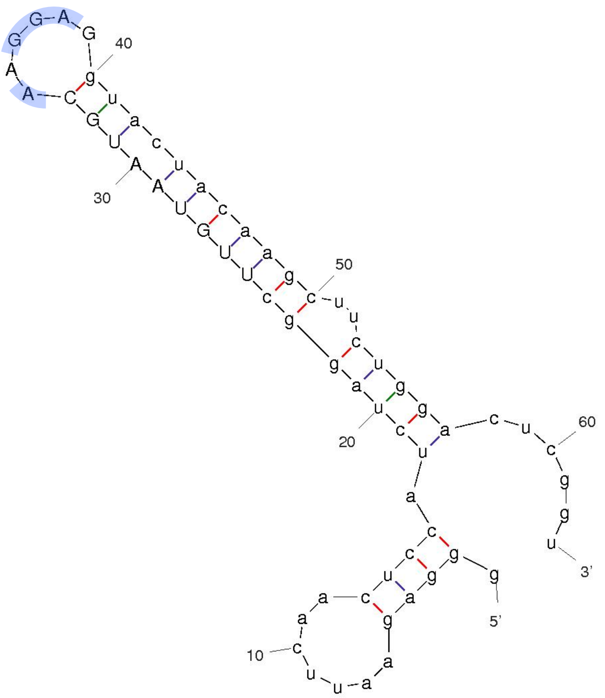

10-4

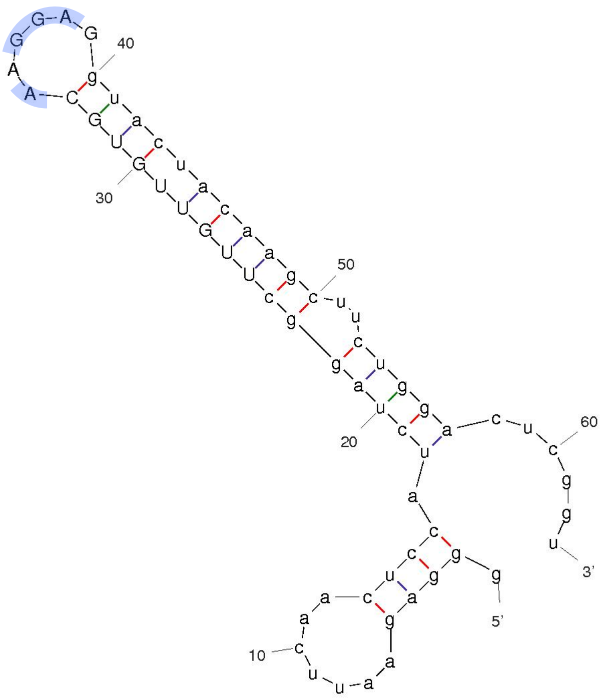

10-27

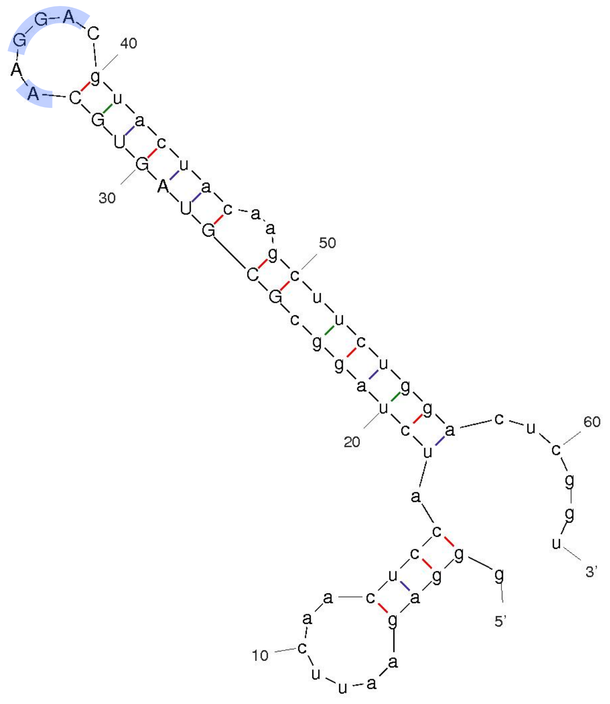

10-37

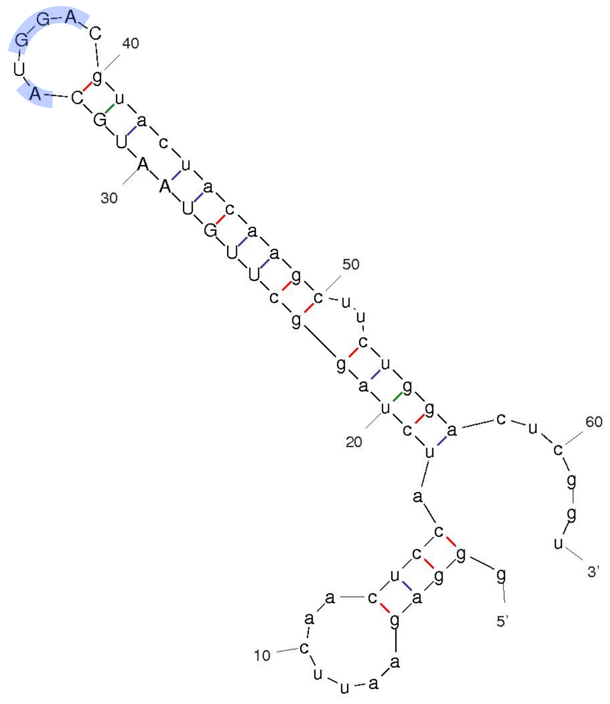

9-26

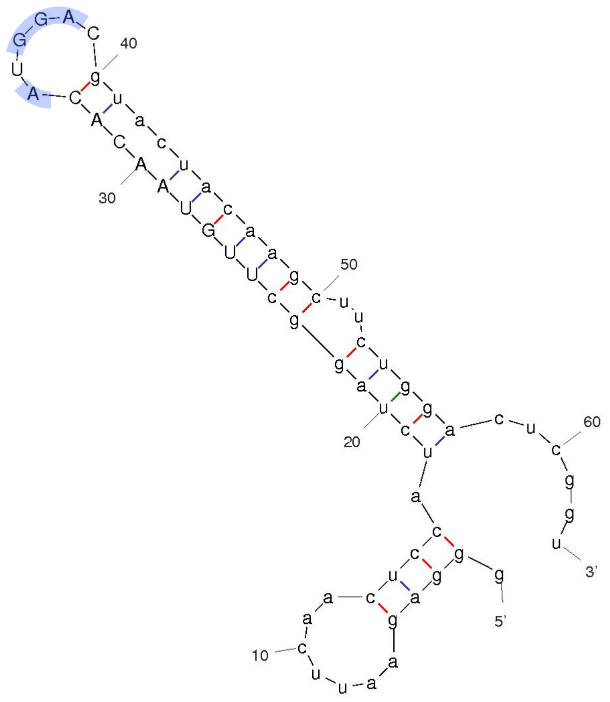

9-29

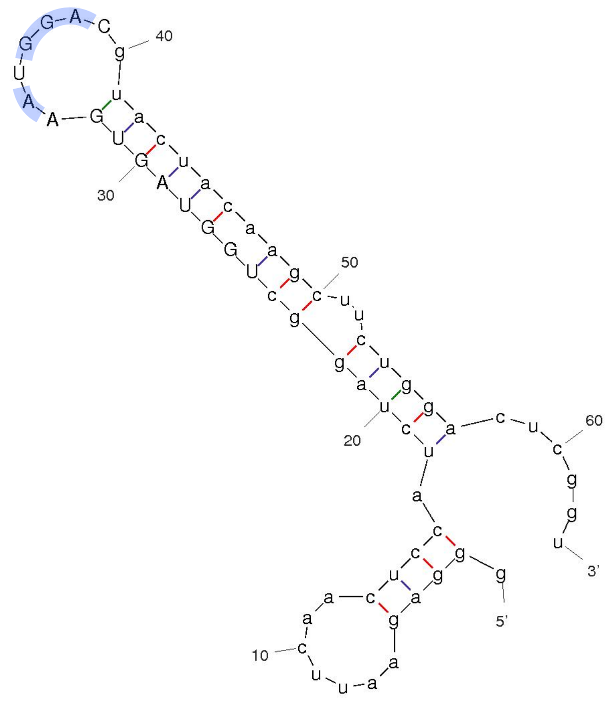

10-31

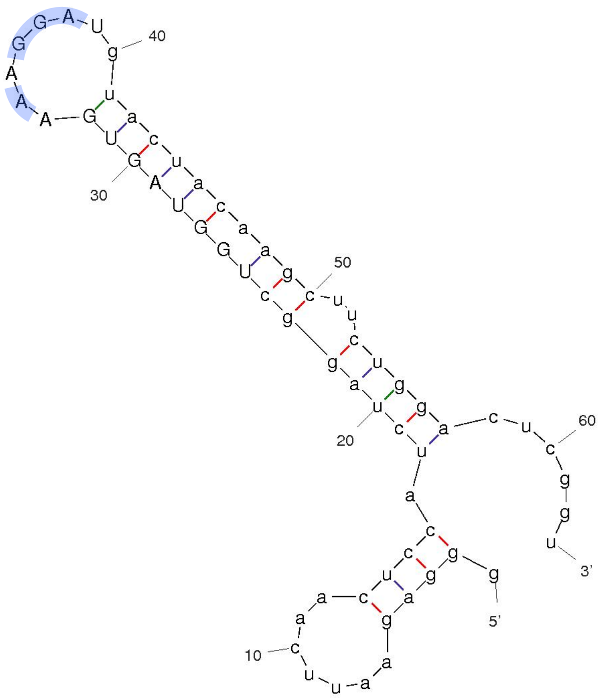

9-39

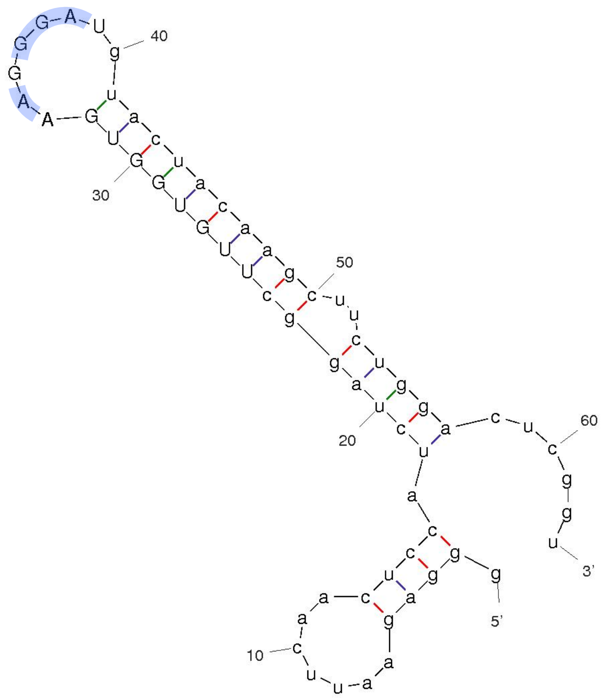

10-2

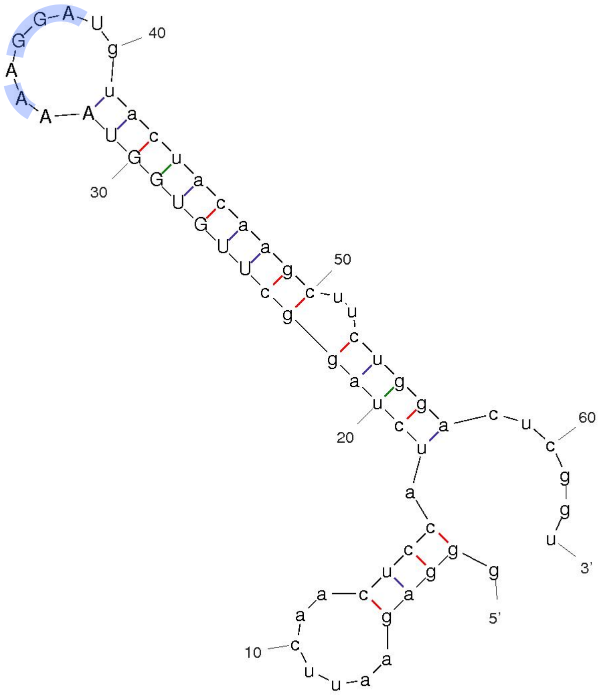

9-37

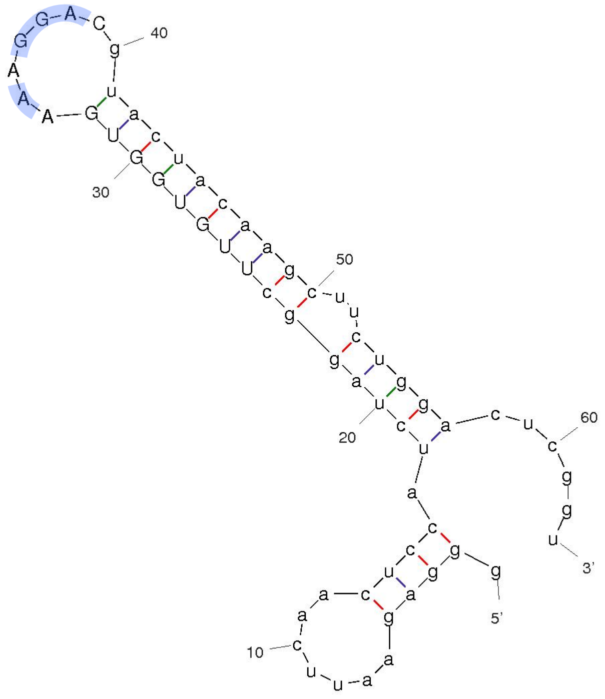

9-5

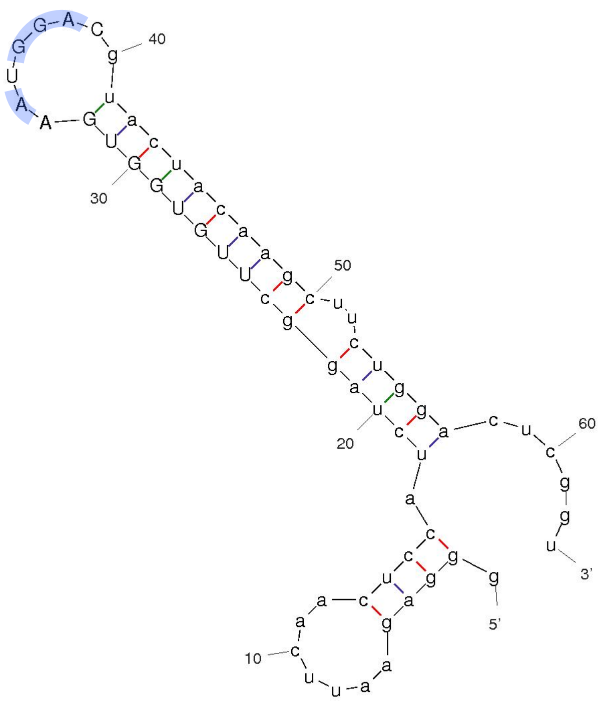

9-31

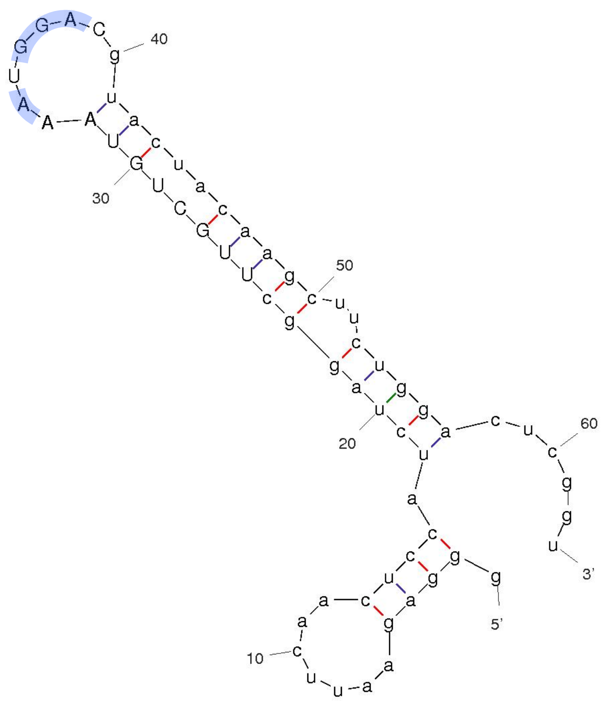

9-2

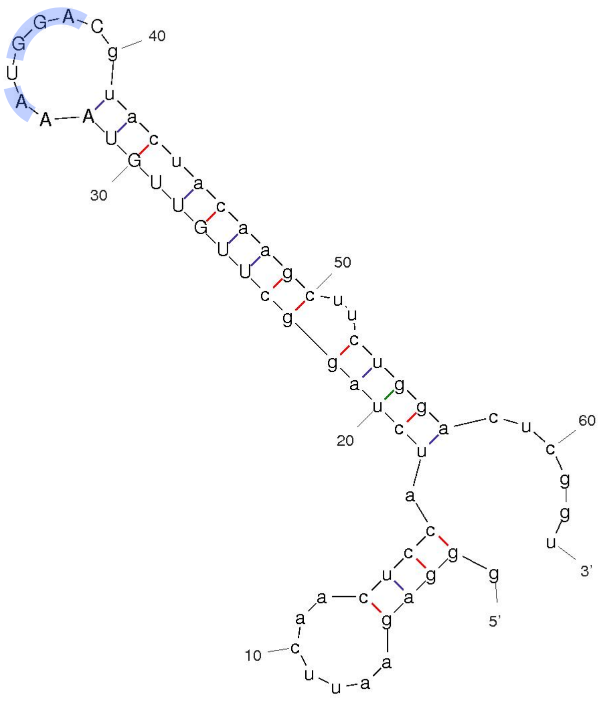

9-19

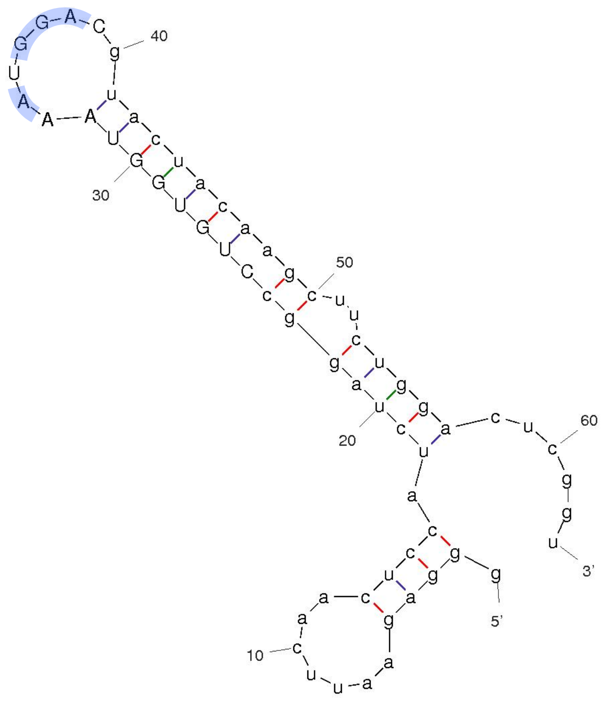

10-20

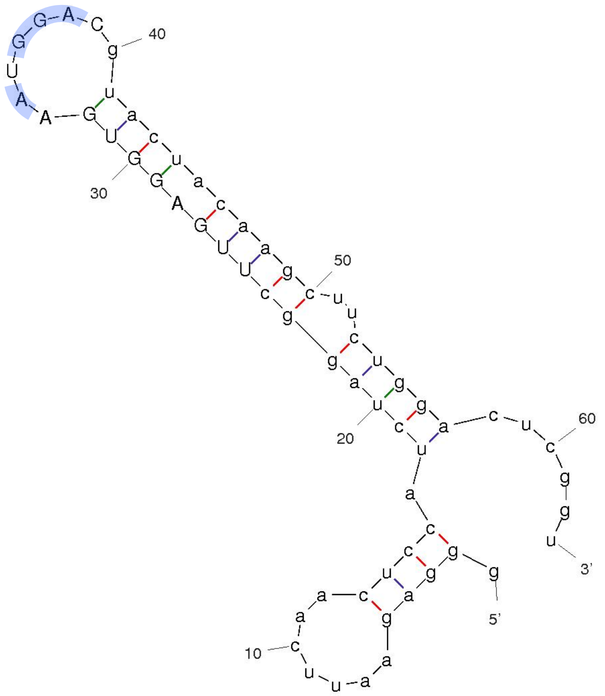

9-3

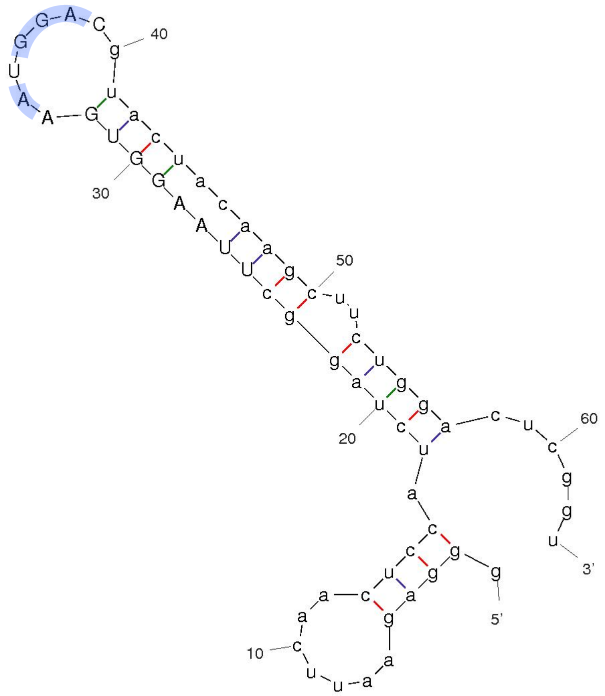

9-24

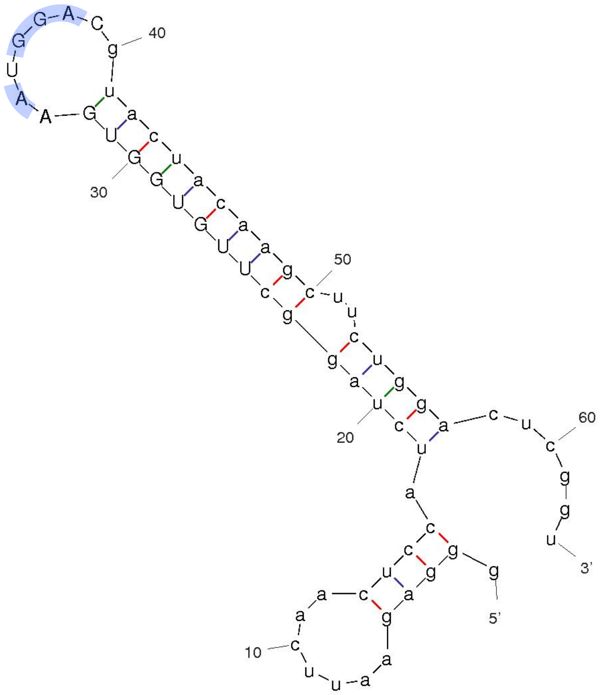

10-26

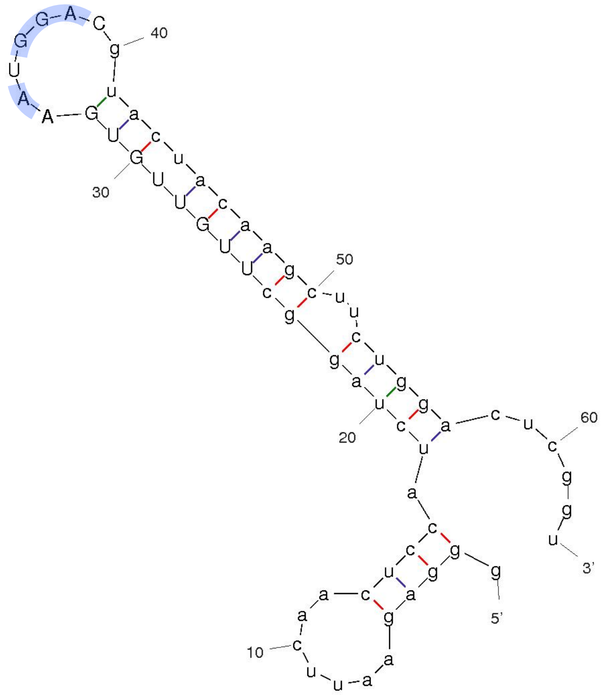

9-23

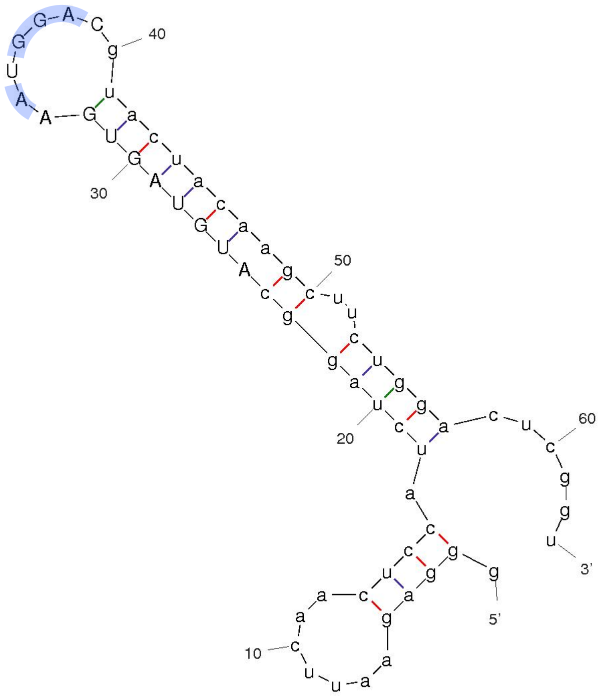

10-16

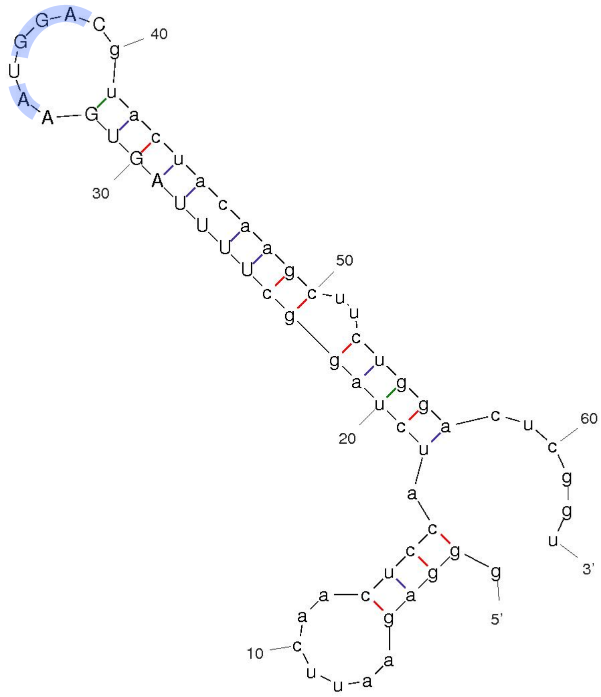

10-24

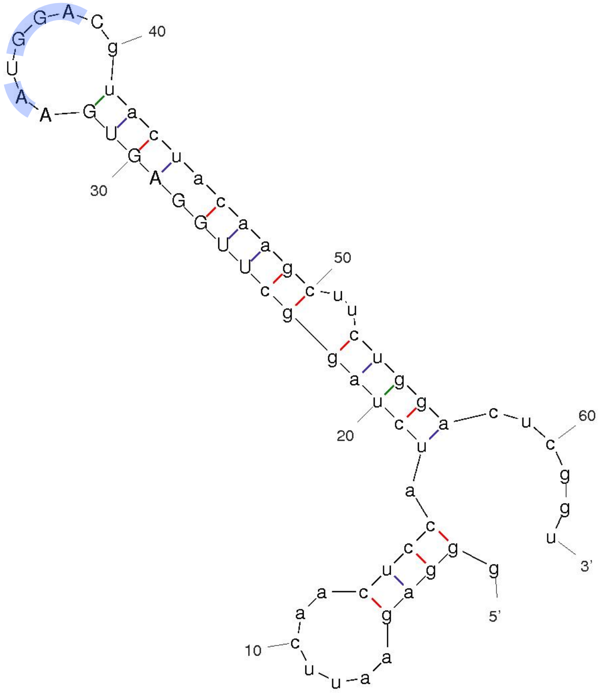

9-33

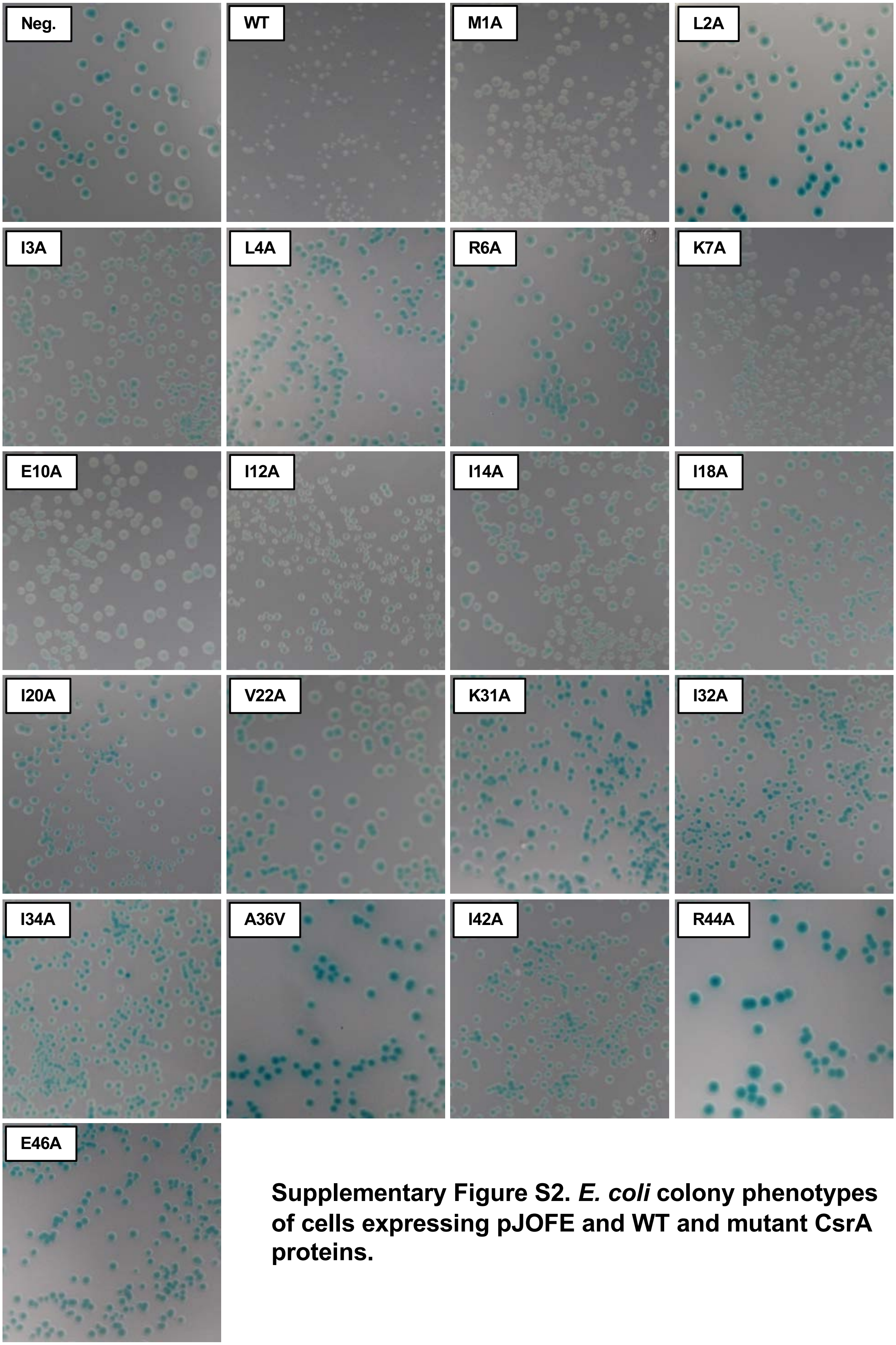

Supplementary Figure S2. ***E. coli*** **colony phenotypes of cells expressing pJOFE and WT and mutant CsrA proteins.**
